# Neutralizing Antibodies to SARS‐CoV‐2 Selected from a Human Antibody Library Constructed Decades Ago

**DOI:** 10.1002/advs.202102181

**Published:** 2021-10-29

**Authors:** Min Qiang, Peixiang Ma, Yu Li, Hejun Liu, Adam Harding, Chenyu Min, Fulian Wang, Lili Liu, Meng Yuan, Qun Ji, Pingdong Tao, Xiaojie Shi, Zhean Li, Teng Li, Xian Wang, Yu Zhang, Nicholas C. Wu, Chang‐Chun D. Lee, Xueyong Zhu, Javier Gilbert‐Jaramillo, Chuyue Zhang, Abhishek Saxena, Xingxu Huang, Hou Wang, William James, Raymond A. Dwek, Ian A. Wilson, Guang Yang, Richard A. Lerner

**Affiliations:** ^1^ Shanghai Institute for Advanced Immunochemical Studies ShanghaiTech University Shanghai 201210 P. R. China; ^2^ School of Life Science and Technology ShanghaiTech University Shanghai 201210 P. R. China; ^3^ Institute of Biochemistry and Cell Biology Shanghai Institutes for Biological Sciences Chinese Academy of Sciences Shanghai 200031 P. R. China; ^4^ University of Chinese Academy of Sciences Beijing 100049 P. R. China; ^5^ Department of Integrative Structural and Computational Biology The Scripps Research Institute La Jolla CA 92037 USA; ^6^ Sir William Dunn School of Pathology University of Oxford Oxford OX1 3RE UK; ^7^ Velox Pharmaceuticals Changzhou 213000 P. R. China; ^8^ ShOx Science Limited Shanghai 200135 P. R. China; ^9^ Department of Biochemistry Oxford Glycobiology Institute South Parks Road Oxford OX1 3QU UK; ^10^ The Skaggs Institute for Chemical Biology The Scripps Research Institute La Jolla CA 92037 USA; ^11^ Department of Chemistry The Scripps Research Institute La Jolla CA 92037 USA

**Keywords:** antibody–antigen interaction, combinatorial antibody library, COVID‐19, neutralizing antibody, SARS‐CoV‐2, somatic hypermutation, variants of concern

## Abstract

Combinatorial antibody libraries not only effectively reduce antibody discovery to a numbers game, but enable documentation of the history of antibody responses in an individual. The severe acute respiratory syndrome coronavirus 2 (SARS‐CoV‐2) pandemic has prompted a wider application of this technology to meet the public health challenge of pandemic threats in the modern era. Herein, a combinatorial human antibody library constructed 20 years before the coronavirus disease 2019 (COVID‐19) pandemic is used to discover three highly potent antibodies that selectively bind SARS‐CoV‐2 spike protein and neutralize authentic SARS‐CoV‐2 virus. Compared to neutralizing antibodies from COVID‐19 patients with generally low somatic hypermutation (SHM), these three antibodies contain over 13–22 SHMs, many of which are involved in specific interactions in their crystal structures with SARS‐CoV‐2 spike receptor binding domain. The identification of these somatically mutated antibodies in a pre‐pandemic library raises intriguing questions about the origin and evolution of these antibodies with respect to their reactivity with SARS‐CoV‐2.

## Graphical abstract

Severe acute respiratory syndrome coronavirus 2 (SARS‐CoV‐2) is the causative agent of the coronavirus disease 2019 pandemic. From a 20‐year‐old, combinatorial library, the authors isolate three neutralizing antibodies with high somatic hypermutations that are involved in antigen recognition, suggesting cross‐reactivity with a related antigen present in the pre‐pandemic‐era.

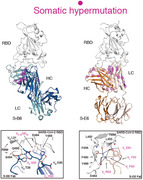

## 1 Introduction

The global spread of severe acute respiratory syndrome coronavirus 2 (SARS‐CoV‐2), a novel coronavirus and cause of the coronavirus disease 2019 (COVID‐19), poses an unprecedented health crisis and was declared a pandemic by the World Health Organization on March 11, 2020.^[^ ^1^ ^]^ As of September 10, 2021, over 222 million individuals have been infected with over 4.5 million deaths (https://covid19.who.int/) with several vaccines or specific antiviral drugs approved. Monoclonal antibodies (mAbs) targeting the viral spike glycoprotein (S) have been shown to have excellent neutralization efficacy in previous treatment of SARS, middle east respiratory syndrome (MERS), and Ebola virus infections as well as in treatment of COVID‐19 as shown by recent clinical data,^[^ ^2^ ^]^ and therefore are of particular interest to combat the current pandemic.^[^ ^3^ ^]^ Since the COVID‐19 outbreak, the spike glycoprotein has been the main target for development of therapeutic mAbs.^[^ ^4^ ^]^ Most neutralizing antibodies (NAbs) bind to the receptor binding domain (RBD) of the S protein,^[^ ^5^ ^]^ although some also bind to the N‐terminal domain.^[^ ^6^ ^]^ NAbs have been derived from multiple sources, including memory B cells from SARS‐CoV‐2 convalescent patients,^[^ ^3^, ^6^, ^7^ ^]^ SARS patients,^[^ ^8^ ^]^ immunized humanized mice that encode the human immunoglobulin repertoire,^[^ ^9^ ^]^ alpaca nanobodies,^[^ ^10^ ^]^ single domain human antibodies from a pre‐established library,^[^ ^11^ ^]^ and phage display antibody libraries.^[^ ^4^, ^12^ ^]^

Antibody generation is an evolutionary process of mutation and selection from the B cell repertoire. The combinatorial antibody library technology allows the same evolutionary process to be performed in vitro as it restores the “fossil record” of an individual's antibody response in a test tube.^[^ ^13^ ^]^ Random coupling of variable heavy chain (V_H_) and variable light chain (V_L_) sequences in single‐chain fragment variable (scFv) libraries greatly expands diversity, thereby allowing for selection of novel antibodies with high binding affinity and neutralization efficacy.^[^ ^14^ ^]^

Here, we report the selection and characterization of three potent SARS‐CoV‐2 antibodies, S‐E6, S‐B8, and S‐D4, from a pre‐pandemic naïve human combinatorial antibody library established in 1999 that target the spike RBD and compete with human angiotensin‐converting enzyme 2 (*h*ACE2) receptor.^[^ ^15^ ^]^ This study provides further evidence that a combinatorial antibody library with an unprecedented diversity can mimic the selection process of natural immunity, permit detection of unexpected, high affinity spike‐targeting antibodies with higher somatic hypermutation (SHM), and allow for selection of binding molecules with chemistries beyond those accessible during in vivo selection.

## 2 Results and Discussion

### 2.1 Selection of Antibodies against SARS‐CoV‐2 Spike RBD

We constructed and overexpressed the SARS‐CoV‐2 spike RBD (S‐RBD) linked to human Fc (*h*Fc) with a thrombin digestion site. After affinity purification, recombinant SARS‐CoV‐2 RBD was biotinylated, immobilized on streptavidin (SA)‐coated magnetic beads, and panned against a combinatorial scFv antibody phage library containing 10^11^ members generated from peripheral blood mononuclear cells of 50 healthy donors in 1999.^[^ ^15^ ^]^ Next‐generation sequencing of the library revealed that 92% of human immunoglobulin heavy chain variable (IGHV) and 89% of the human immunoglobulin lambda variable (IGLV) and kappa variable (IGKV) germline genes were covered, when aligned to the international ImMunoGeneTics (IMGT) database (Figure S1a, Supporting Information), enabling screening of antibodies encoded by diverse germlines. Of note, by analyzing ≈400 000 sequences in the library (209 000 IGHV sequences, 93 000 IGKV sequences, and 87 000 IGLV sequences), the selected antibodies displayed low SHM levels as expected for a naïve library, with over 70%, 61%, and 80% sequences of IGHV, IGKV, and IGLV, respectively, having no more than 3 amino acid mutations (Figure S1b–d, Supporting Information).

**Figure 1:**
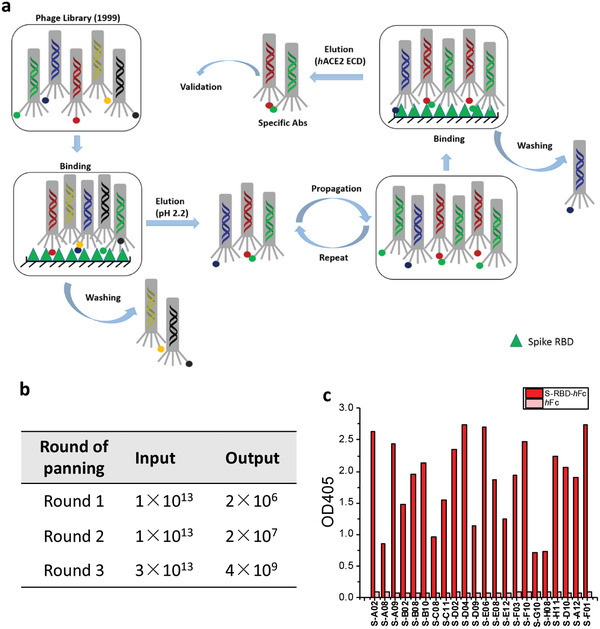
Selection of scFv antibodies targeting SARS‐CoV‐2 spike protein. a) Workflow of the panning process against S‐RBD. b) Input and output versus panning round for the antigen S‐RBD‐*h*Fc during three rounds of screening. c) Phage ELISA results of 22 unique antibodies with positive readouts (OD_405_ ratio S‐RBD‐*h*Fc/*h*Fc > 2).

In the first two rounds of panning, a pH 2.2 glycine‐HCl solution was used to elute antibody‐displaying phagemids bound to S‐RBD. To enrich for antibodies that compete with *h*ACE2, a “function‐guided enrichment” strategy was used in the third round, where recombinant *h*ACE2 ectodomain (*h*ACE2‐ECD) protein was used to elute S‐RBD binding phagemids (**Figure** **1**a; Figure S1, Supporting Information). After three rounds of panning, SARS‐CoV‐2 RBD‐reactive phages were enriched (Figure 1b). Pooled phages were tested for binding to the RBD‐*h*Fc by phage ELISA (Figure 1c). Positive phages were sent for Sanger sequencing and 22 unique clones were identified by sequence analysis.

### 2.2 Selected Anti‐S‐RBD Antibodies Retain Binding to Full‐Length Spike

**Figure 2:**
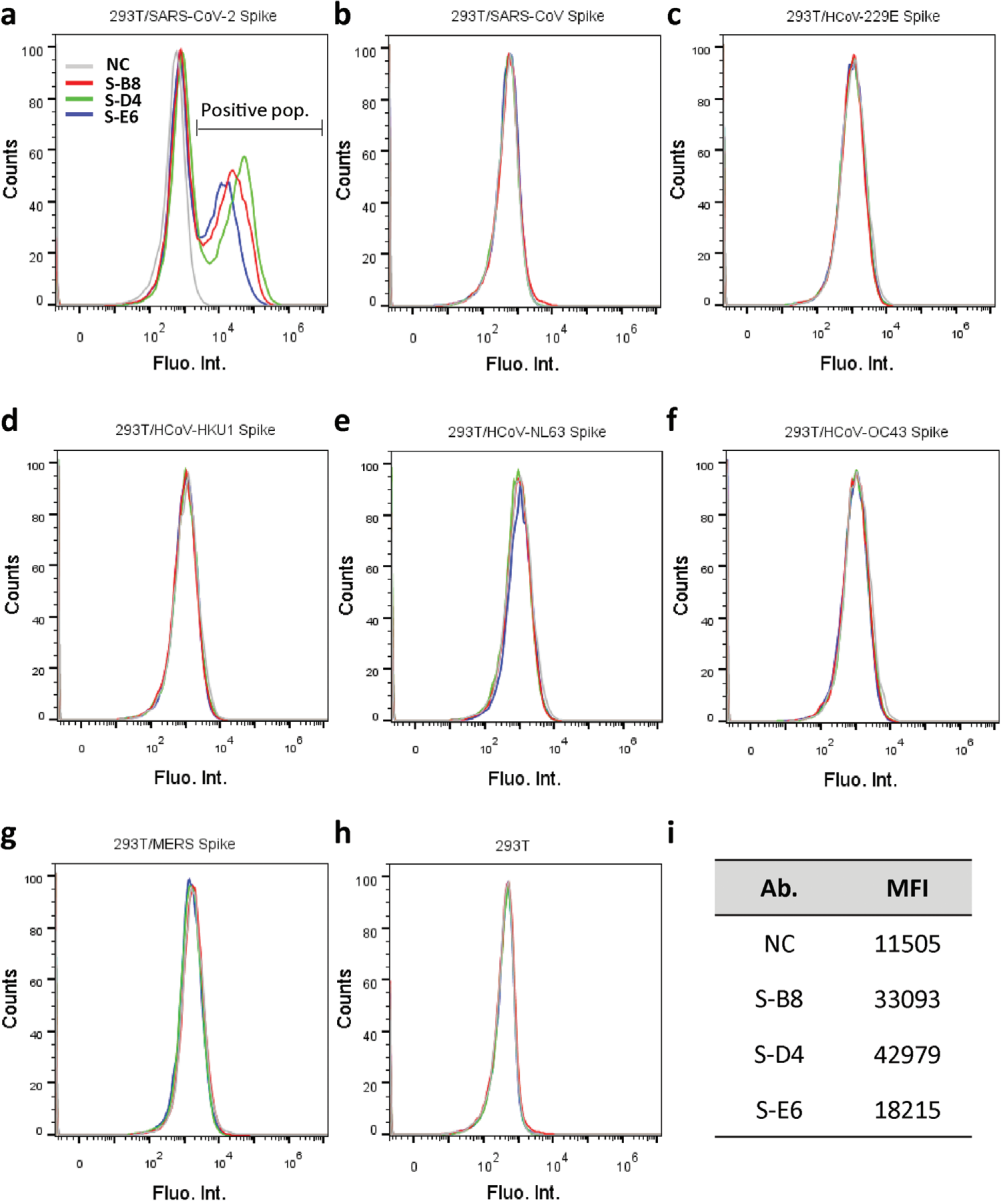
Analysis of antibody binding to cell surface‐expressed trimeric spike protein. a) HEK293T cells transfected with expression plasmid encoding the full‐length spike of SARS‐CoV‐2 were incubated with purified IgG4 antibody and stained with PE labeled anti‐human IgG4 Fc secondary antibody, then analyzed by FACS. Positive binding cell populations were labeled as positive pop. b–g) FACS of antibodies binding to SARS‐CoV spike, HCoV‐229E spike, HCoV‐HKU1 spike, HCoV‐NL63 spike, HCoV‐OC43 spike, and MERS spike. h) FACS of antibodies binding to non‐transfected HEK293T cells. Cells stained with only secondary antibody were used as negative control (NC). i) Mean fluorescent intensity (MFI) of antibodies for SARS‐CoV‐2 spike binding, that is, positive population area in (a).

All 22 scFv antibodies were then converted to full‐length mAbs by cloning into a human IgG4e1(S228P) vector. The constructed antibodies were expressed in suspension‐adapted HEK293F cells with a secretion signal followed by clarification of the cultured medium. The resultant supernatants were evaluated by their binding to cell‐surface expressed SARS‐CoV‐2 spike (Figure S1e, Supporting Information) and competition with *h*ACE2‐ECD (Figure S1f, Supporting Information). Three of the 22 antibodies exhibited the best performance with respect to binding and competition. Antibodies S‐B8, S‐D4, and S‐E6 were then purified to homogeneity with yields of 8.1, 9.6, and 17 mg L^−1^, respectively, whereas the yield for SARS‐CoV‐2 RBD‐*h*Fc (IgG1) was 58 mg L^−1^ (Figures S2a–c and S3a, Supporting Information). A thermal stability assay using nano differential scanning fluorimetry showed decent melting temperatures (*T*_m_) of 67.8, 55.7, and 66.3 °C for S‐B8, S‐D4, and S‐E6, respectively (Figure S2d–f, Supporting Information). Since S‐E6 is the most potent neutralizing antibody with no known auto‐reactivity, we further tested the thermal stability of S‐E6 by an HPLC‐SEC assay. Aliquots of S‐E6 were incubated at 45 °C for up to 72 h and analyzed by size‐exclusion‐high‐performance liquid chromatography (SEC‐HPLC). S‐E6 showed high stability under different incubating times despite a small fraction of aggregates appears after incubating for 48 h at 45 °C (Figure S2g,h, Supporting Information). These data suggest further antibody engineering may be needed for any therapeutic development. To characterize interactions between the anti‐S‐RBD antibodies and full‐length spikes, HEK293T cells were transiently transfected with either SARS‐CoV‐2 spike‐P2A‐EGFP or other coronavirus spike‐P2A‐EGFP. Flow‐cytometry (FACS) showed that all three antibodies in full‐length‐IgG4 format retained their ability to bind full‐length SARS‐CoV‐2 spike (**Figure** **2**a,i) with no cross‐reactivity to other human coronavirus (HCoV) spikes, including SARS‐CoV (Figure 2b), HCoV‐229E (Figure 2c), HCoV‐HKU1 (Figure 2d), HCoV‐NL63 (Figure 2e), HCoV‐OC43 (Figure 2f), MERS‐CoV (Figure 2g), or with non‐transfected cells (Figure 2h).

### 2.3 Antibody Binding and Competition with *h*ACE2‐ECD to SARS‐CoV‐2 RBD

**Figure 3:**
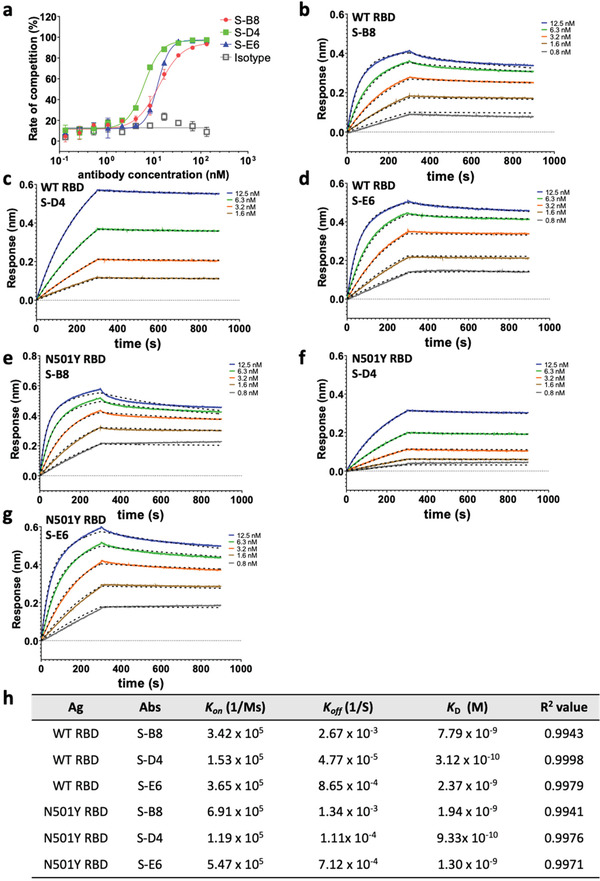
Competitive ELISA of antibodies with *h*ACE2 and binding kinetics to the spike protein. a) The three antibodies were titrated for competition with *h*ACE2‐ECD for binding to S‐RBD and the fitting curves are shown (*n* = 3). b–d) Binding kinetics with wild‐type (WT) S‐RBD were measured by biolayer interferometry (BLI). Biotinylated S‐RBD was loaded to the streptavidin (SA) biosensor for detection of binding kinetics with S‐B8 (b) and S‐E6 (d), while S‐RBD amine coupled to amine reactive second‐generation (AR2G) sensor was utilized for S‐D4 (c), with detection on an Octet. All curves were fitted with a 1:2 binding model with globally linked *R*_max_ using the Data Analysis software (ForteBio). The superimposed dashed lines indicate the model fitting curves. Binding kinetics with N501Y S‐RBD was measured by biolayer interferometry (BLI) as above, with the binding and fitting curves of e) S‐B8, f) S‐D4 and g) S‐E6 shown. h) The association‐rate (*k*_on_), dissociation‐rate (*k*_off_), dissociation constant (*K*_D_), and *R* ^2^ value for fitting of the three competitive antibodies to WT S‐RBD and N501Y S‐RBD are shown. Ag: antigen, Abs: antibodies.

To assess the neutralization potential of the mAbs, we investigated their ability to compete with *h*ACE2‐ECD for S‐RBD binding by ELISA.^[^ ^12^, ^16^ ^]^ S‐B8, S‐D4, and S‐E6 IgG all competed strongly with *h*ACE2‐ECD in a dose‐dependent manner, with IC_50_ values of 12.9 ± 1.5, 7.1 ± 0.4, and 12.2 ± 0.7 nm, respectively (**Figure** **3**a). Competition between S‐E6 and S‐B8 (Figure S3b, Supporting Information) or S‐D4 (Figure S3c, Supporting Information) for binding to S‐RBD was also observed, indicating epitope overlap between the three antibodies. Kinetic parameters of on‐rate (*k*_on_), off‐rate (*k*_off_), and dissociation constant (*K*_D_) for these antibodies were then determined by biolayer interferometry (BLI) using a 1:2 fitting model (Figure 3b–d,h). S‐B8, S‐D4, and S‐E6 IgG exhibited apparent *K*_D_ values of 7.79, 0.31, and 2.37 nm, respectively. S‐D4 demonstrated the highest binding affinity due to a slower off‐rate (Figure 3h). To further assess the binding kinetics of a monovalent interaction between antibody and antigen using a 1:1 fitting model, antibody Fabs were used for the kinetics assay. The Fabs also showed strong binding to SARS‐CoV‐2 RBD with *K*_D_ values of 15.4, 5.18, and 11.4 nm for S‐B8, S‐D4, and S‐E6 Fab, respectively (Figure S4a–d, Supporting Information). The differences in the IgG versus Fab *K*_D_ values could be due to some contribution of IgG avidity effects, which have been observed for binding of some SARS‐CoV‐2 antibodies where the two Fabs on the IgG can bind to two RBDs on the same spike protein and would lead to 1:2 binding compared to monovalent 1:1 binding for the Fab.^[^ ^17^ ^]^ IgG avidity effects have also been observed for antibody IgG binding between spike trimers.^[^ ^18^ ^]^ Compared to the wild‐type spike RBD, binding affinities of the three antibody IgGs to the RBD with N501Y mutation that is observed in the alpha variant of concern B.1.1.7^[^ ^19^ ^]^ were greater with *K*_D_ values of 1.94, 0.93, and 1.30 nm for S‐B8, S‐D4, and S‐E6, respectively (Figure 3e–h; Figure S4e–j, Supporting Information).

However, the binding abilities of all three antibodies were greatly affected by the E484K mutation in RBD that emerged in beta and gamma variants of concern B.1.351^[^ ^20^ ^]^ and P.1,^[^ ^21^ ^]^ as shown by the dramatically decreased binding signals to E484K+N501Y RBD (Figure S4k,m,o, Supporting Information). For S‐B8, binding to K417N+E484K+N501Y RBD‐His (Figure S4l, Supporting Information) is slightly greater than to E484K+N501Y RBD‐His (Figure S4k, Supporting Information), whereas S‐D4 and S‐E6 exhibit similar weak binding to K417N+E484K+N501Y RBD‐His (Figure S4n,p, Supporting Information).

We also tested natural mutants of SARS‐CoV‐2 spike proteins that have been clinically associated with more severe illness and longer hospital stays, as well as the key amino‐acid mutations of spike proteins in circulating variants such as alpha and beta by FACS assay. We evaluated three mutant spike proteins (Figure S5a–c, Supporting Information), that is, D215H (mut 1), S247R (mut 2), and D614G (mut 3), found in patients requiring treatment in an intensive care unit,^[^ ^22^ ^]^ spike mutant N439K+D614G found in mink (mut 4; Figure S5d, Supporting Information),^[^ ^23^ ^]^ alpha variant spike (Figure S5e, Supporting Information), key mutation N501Y+D614G (mut 5; Figure S5f, Supporting Information) that enhances RBD affinity to *h*ACE2 in rapidly spreading variants,^[^ ^24^ ^]^ E484K+N501Y+D614G (mut 6; Figure S5g, Supporting Information) found in beta and gamma variants, K417N+E484K+N501Y+D614G (mut7; Figure S5h, Supporting Information) in the beta variant, and K417N+N501Y+D614G (mut 8; Figure S5i, Supporting Information). All displayed positive binding by FACS to S‐B8, S‐D4, and S‐E6. By analyzing the mean fluorescent intensity (MFI) of each antibody to each mutant (Figure S5j, Supporting Information), we found that binding of S‐B8 to mut 6 (to 18% of mut 3; Figure S5g, Supporting Information) and mut7 (to 14.7% of mut3; Figure S5h, Supporting Information) decreased significantly. Similarly, S‐D4 binding to mut 6 and mut 7 decreased to 33.2% and 35.6% of mut 3, respectively (Figure S5j, Supporting Information). However, the MFI of S‐E6 to mut 6 and mut 7 was similar to that of mut 3 (Figure S5j, Supporting Information) that differs from the BLI data (Figure S4o,p, Supporting Information) and suggests some differences are detected on the spike expressed on cells versus that on the S‐RBD for this particular antibody.

### 2.4 Inhibition of Cell–Cell Fusion Induced by SARS‐CoV‐2 Spike and *h*ACE2

**Figure 4:**
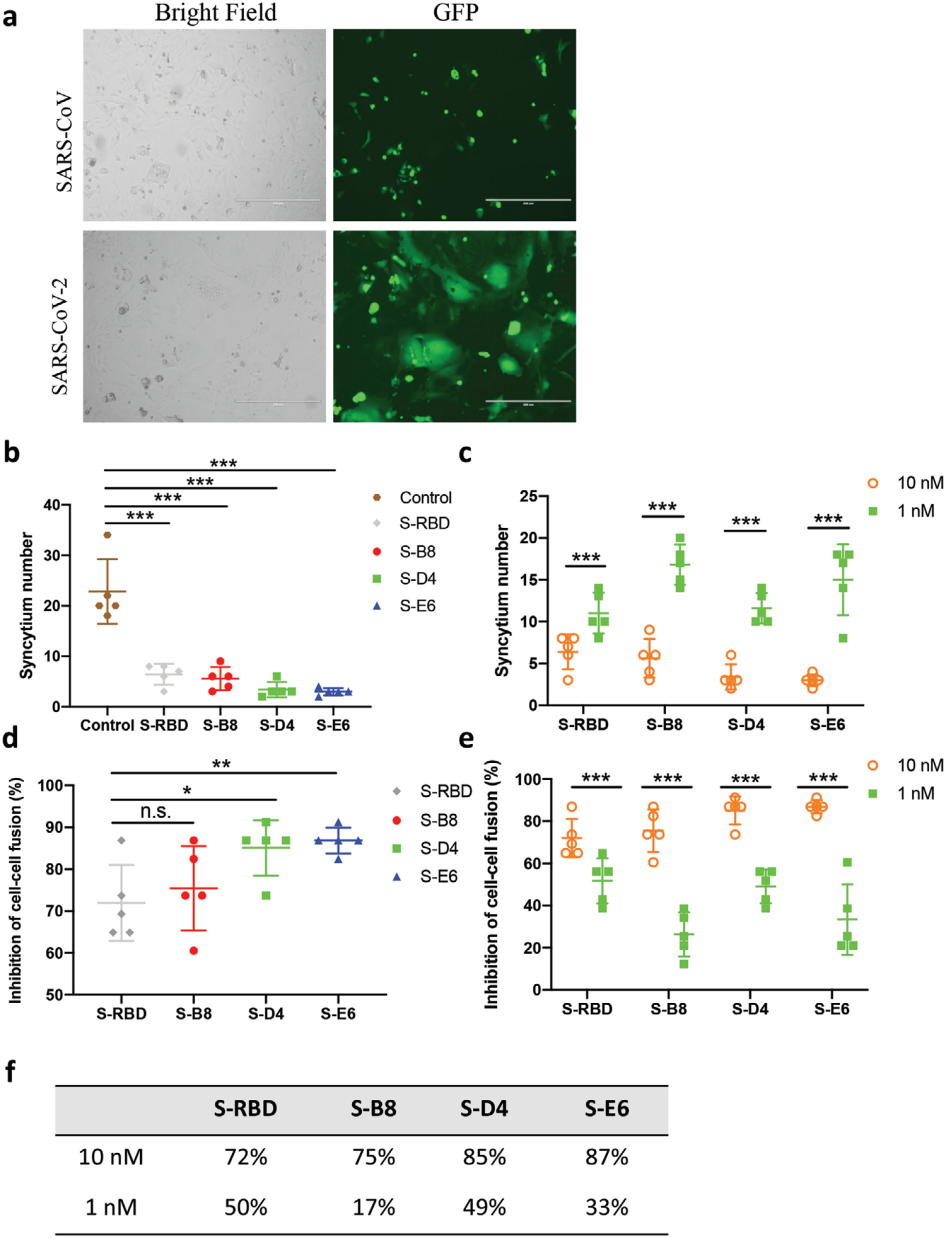
Inhibition of syncytium formation by the antibodies. a) Representative images of SARS‐CoV‐2 and SARS‐CoV spike‐mediated syncytium formation with *h*ACE2 expressing cells 48 h after co‐culture. b,d) Syncytium number calculation and inhibition rates when treated with 10 nm of *h*ACE2 competitive antibodies are shown. S‐RBD was used as the positive control (*n* = 5). c,e) Syncytium number and inhibition after treatment with antibodies and S‐RBD at different concentrations are shown (*n* = 5). f) The inhibition rates at 10 and 1 nm are summarized. Bars = 400 µm. Error bars indicate SD, ^*^ *p* < 0.05, ^**^ *p* < 0.01, ^***^ *p* < 0.001, determined by Student's *t*‐test in GraphPad Prism software.

The S2 subunit of the SARS‐CoV‐2 spike mediates membrane fusion in *h*ACE2 expressing cells and is essential for virus infection. *h*ACE2 binding to SARS‐CoV‐2 is stronger than to the SARS‐CoV spike (*K*_D_ of 4.7 and 32 nm, respectively).^[^ ^25^ ^]^ To test whether these antibodies could inhibit spike‐mediated membrane fusion of cells, we established a cell–cell fusion assay using Vero cells overexpressing *h*ACE2 as target cells, SARS‐CoV‐2 spike‐P2A‐EGFP transient transfected HEK293F cells as effector cells, and SARS‐CoV spike‐P2A‐EGFP cells as a negative control. Spike‐expressing HEK293F cells were mixed with S‐B8, S‐D4, or S‐E6 at 10 or 1 nm just before adding to the Vero cells and syncytium formation observed 6 h later. The SARS‐CoV‐2 spike induced significant cell–cell fusion as manifested by formation of larger EGFP positive cells, whereas the SARS‐CoV spike barely induced syncytium formation (**Figure** **4**a). All three antibodies inhibited cell–cell fusion by SARS‐CoV‐2 at both 10 and 1 nm with 10 nm being significantly more potent (Figure 4b,c,f). At 10 nm, S‐D4 and S‐E6 exhibited over 80% inhibition of cell–cell fusion, which was slightly greater than recombinant S‐RBD; S‐D4 and S‐E6 were also more potent than S‐B8 at 1 and 10 nm (Figure 4d,e).

### 2.5 Inhibition of SARS‐CoV‐2 Pseudovirus and Authentic Virus

**Figure 5:**
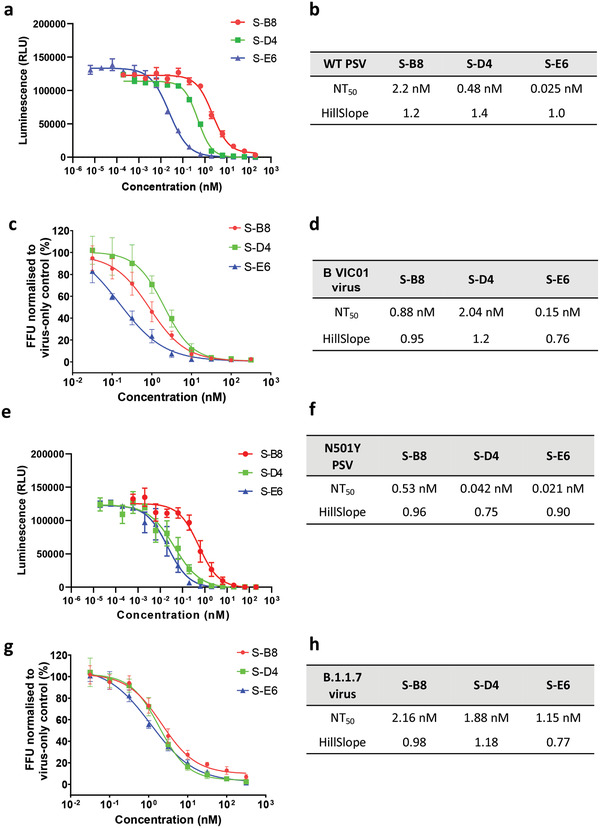
Neutralization assay for the *h*ACE2 competitive antibodies. a) Neutralization ability of the three *h*ACE2 competitive antibodies to WT SARS‐CoV‐2 pseudovirus was tested and fitted (*n* = 3). c) A microneutralization assay was adopted for testing of the three antibodies (*n* = 4). b,d) NT_50_ and HillSlope for each antibody on authentic SARS‐CoV‐2 are summarized. e,f) Neutralization ability of the three *h*ACE2 competitive antibodies to SARS‐CoV‐2 N501Y+D614G mutant pseudovirus was tested and fitted (*n* = 3), NT_50_ and HillSlope are shown. g) Neutralization of three antibodies to real virus alpha variant (*n* = 4) and h) NT_50_ and HillSlope for each antibody are shown. Error bars indicate SD, GraphPad Prism software was used for fitting and NT_50_ determination.

To test neutralization against SARS‐CoV‐2 virus, we first assessed the antibodies in a pseudovirus (PSV) infection assay. Pseudotyped particles were pre‐incubated with S‐B8 and S‐D4 (from 200 nm to 200 fm) and S‐E6 (200 nm to 6.3 fM), followed by infection of HEK293T/*h*ACE2 cells. Luciferase activity resulting from infection was determined at 60 h post transfection. All three antibodies showed potent neutralization against wild‐type SARS‐CoV‐2 PSV infection in a dose‐dependent manner that went to completion. The NT_50_ values of S‐B8, S‐D4, and S‐E6 in the PSV neutralization were determined to be 2.2 ± 0.2, 0.48 ± 0.03, and 0.025 ± 0.002 nm, respectively using four‐parameter non‐linear regression fitting model (**Figure** **5**a,b). We next tested antibody neutralization of authentic SARS‐CoV‐2 virus [BetaCoV/Australia/VIC01/2020; GenBank MT007544.1 (Victoria/01/2020), B VIC01]. Twenty hours after infection, intracellular virus was visualized and quantitated as percent infectivity. All three antibodies were capable of fully blocking infection by authentic virus B VIC01 with NT_50_ values for S‐B8, S‐D4, and S‐E6 of 0.88 ± 0.14, 2.04 ± 0.31, and 0.15 ± 0.06 nm, respectively (Figure 5c,d).

Due to the emergence of the N501Y mutation in the RBD of the alpha variant, we also tested the neutralization abilities of the three antibodies to SARS‐CoV‐2 spike N501Y+D614G PSV. All three antibodies appeared to display better neutralizing efficacy than to wild‐type PSV. The NT_50_ values of S‐B8, S‐D4, and S‐E6 in N501Y+D614G spike PSV neutralization were determined to be 0.53 ± 0.09, 0.042 ± 0.008, and 0.021 ± 0.003 nm, respectively (Figure 5e,f). Tests on authentic SARS‐CoV‐2 alpha variant showed that all three antibodies maintain neutralization ability (Figure 5g), with NT_50_ values for S‐B8, S‐D4, and S‐E6 of 2.16 ± 0.28, 1.88 ± 0.20, and 1.15 ± 0.14 nm, respectively (Figure 5h). Compared to their ability to neutralize wild‐type SARS‐CoV‐2 authentic virus, the ability of S‐B8 and S‐E6 to neutralize the alpha variant decreased by ≈2.4‐ and 7.6‐fold, respectively (Figure 5d,h). However, S‐D4 showed slightly better potency.

As one might expect from the decrease in their binding affinity to E484K and K417N+E484K variants, all three antibodies showed dramatically decreased neutralization ability when tested against the K417N+E484K+N501Y spike mutant PSV (Figure S6a, Supporting Information). Antibody concentrations of 200 nm were still not sufficient to completely block PSV infection. A similar decrease in neutralizing ability was observed for all three antibodies when tested against the authentic beta variant (Figure S6b, Supporting Information). However, S‐D4 did show weak neutralization at high concentrations (>50 nm) against the beta variant.

### 2.6 S‐B8 and S‐E6 Bind the RBD and Sterically Block ACE2 Binding

**Figure 6:**
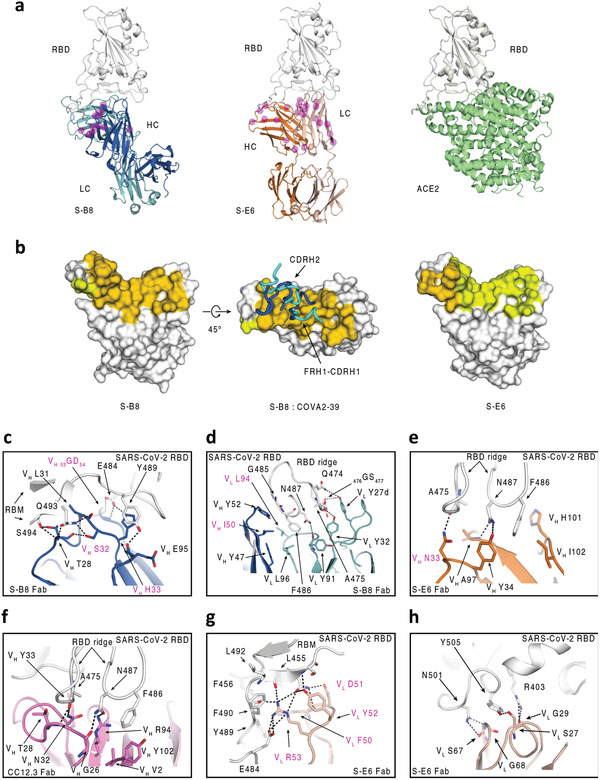
Structural characterization of S‐B8 and S‐E6 with SARS‐CoV‐2 RBD. Crystal structures are shown in ribbon representation with residues of interest in stick mode. The epitope surface on the RBD involved in interaction with the heavy and light chains of the antibodies are in orange and yellow, respectively. S‐RBD is shown in white, S‐B8 in blue and light blue for heavy and light chains, S‐E6 heavy and light chains in orange and pink, and *h*ACE2 in green. SHM residues are shown as semi‐transparent magenta spheres and highlighted with magenta labels in (c–h). a) Structure comparison of S‐B8 and S‐E6 compared to *h*ACE2 binding to the RBD in the same relative view. b) Surface representation of S‐RBD epitope residue interactions with S‐B8 and S‐E6. FRH1‐CDRH1 and CDRH2 from both S‐B8 (blue) and COVA2‐39 (cyan, PDB 7JMP) are shown for comparison. c) S‐B8 CDRH1 and CDRH2 interaction with RBD. d) Interaction between S‐B8 and RBD ridge. e) Interaction between S‐E6 and RBD ridge. f) Comparison to IGHV3‐53 binding mode A. CC12.3 (pink for heavy chain and light pink for light chain) in complex with SARS‐CoV‐2 RBD (PDB 6XC7) illustrating the hydrogen bonding between the _32_NY_33_ motif and S‐RBD. g) Interaction between S‐E6 and RBM mid‐region. h) Interaction between S‐E6 and RBM on the opposite side of the S‐RBD ridge.

To elucidate the molecular recognition of S‐B8 and S‐E6 for SARS‐CoV‐2 RBD, X‐ray structures of Fab+RBD complexes were determined to 2.25 and 2.70 Å, respectively (Table S1, Supporting Information). Fab S‐B8 and S‐E6 bind the receptor binding site (RBS) with different approach angles (**Figure** **6**a) and sterically compete with ACE2 for RBD binding, consistent with the competition assay (Figure 3a). S‐B8 interacts mainly using its heavy chain, which contributes 73% of the buried surface area (BSA, 737 of 1010 Å^2^) (Figure 6b) and 12 of 16 polar contacts (Table S2, Supporting Information). S‐E6 predominately uses its light chain, which contributes 63% of the BSA (530 of 847 Å^2^) and 16 of 19 polar contacts (Table S2, Supporting Information). Light‐chain dominant interactions are less common in antibodies.^[^ ^26^ ^]^

IgBLAST analysis^[^ ^27^ ^]^ suggests S‐B8 is derived from IGHV3‐66, a germline that is highly similar to IGHV3‐53 (Figure S7, Supporting Information). A previous report showed that IGHV3‐53 antibodies isolated from convalescent patients, with minimal SHM and high potency, have two key germline motifs in CDRH1 and CDRH2 that are primarily used for recognition of SARS‐CoV‐2 RBD, namely _32_NY_33_ and _53_SGGS_56_.^[^ ^28^ ^]^ In addition, two very distinct binding modes (A and B) are observed for IGHV3‐53/3‐66 antibodies depending on CDRH3 length as reported previously.^[^ ^28^, ^29^ ^]^ These particular germline‐encoded antibodies with short CDRH3 loops are more likely to bind SARS‐CoV‐2 RBD in mode A.^[^ ^28^, ^30^ ^]^ However, in S‐B8, _32_NY_33_ in CDRH1 is mutated to _32_SH_33_ and _53_SCGS_56_ (_53_TGGT_56_ in COVA2‐39, an IGHV3‐53 antibody derived from convalescent patient^[^ ^6b^ ^]^) in CDRH2 to _53_GDGN_56_ (Figures S8 and S9, Supporting Information). Intriguingly, CDRH1 and CDRH2, as well as FRH1 of S‐B8 still bind to a similar region on SARS‐CoV‐2 RBD to that of binding mode B (Figure 6b).^[^ ^28b^ ^]^ The _32_SH_33_ in S‐B8 is part of a type I beta‐turn (Figure 6c). V_H_ S32 side chain hydrogen bonds with RBD Q493 and the V_H_ T28 carbonyl oxygen. The V_H_ H33 imidazole forms a salt bridge with RBD E484 and a *π*–*π* interaction with Y489. The V_H 53_GD_54_ backbone in CDRH2 also forms two hydrogen bonds with E484, and V_H_ T28 and L31 make four hydrogen bonds with Q493 and S494 (Figure 6c; Table S2, Supporting Information). F486 in the S‐RBD ridge region is buried in a hydrophobic pocket (V_H_ W47, V_H_ I50, V_L_ Y91, V_L_ L94, and V_L_ L96) between the heavy and light chains, while _485_GF_486_ and _476_GS_477_ on the RBD ridge interact with V_H_ Y52 and V_L_ Y27d via *π*–*π* interactions (Figure 6d). Of note, F486 is also buried in a pocket at the heavy‐light chain interface in COVA2‐39, which is also an IGHV3‐53 antibody, as well as other antibodies that bind in the RBS‐B mode.^[^ ^30^ ^]^ Altogether, 19 of 29 S‐B8 epitope residues are shared with 19 of 21 COVA2‐39 epitope residues, with 16 corresponding to ACE2 binding residues (Figure S10, Supporting Information).

### 2.7 S‐E6 Interaction with SARS‐CoV‐2 RBD

S‐E6 is an IGHV4‐31 antibody. Interestingly, SHM introduces a _33_NY_34_ sequence in a similar position to the _32_NY_33_ motif in CDRH1 in IGHV3‐53/3‐66 antibodies (Figure S8, Supporting Information) that interact with the same RBD site but in a different orientation compared to _32_NY_33_ of IGHV3‐53 binding mode A.^[^ ^30^ ^]^ Nevertheless, V_H_ N33 still hydrogen bonds with RBD A475 carbonyl (Figure 6e), as does V_H_ N32 of IGHV3‐53 in binding mode A (Figure 6f). V_H_ Y34 and V_H_ A97 form two hydrogen bonds with N487 of the S‐RBD (Figure 6e), which differ from Y33 in IGHV3‐53 antibodies (Figure 6f). F486, along with N487, interact with a hydrophobic pocket formed by V_H_ Y34, A97, H101, and I102 of S‐E6 and also make *π*–*π* and cation–*π* interactions (Figure 6e). However, the S‐E6 light chain contributes the majority of the buried surface with the RBD. CDRL2 _50_FDYR_53_ interact with the receptor binding motif (RBM) via multiple polar interactions (eight hydrogen bonds and three salt bridges) to E484, F490, L492, Q493, and S494 (Figure 6g; Table S2, Supporting Information). Moreover, V_L_ F50 interacts with a nearby hydrophobic patch formed by L455, F456, and Y489 (Figure 6g), and V_L_ S27, G29, S67, and G68 form five hydrogen bonds with R403, N501, and Y505 on the other side of the RBS ridge (Figure 6h; Table S2, Supporting Information). However, residue 501 is located at the edge of S‐E6 epitope site with space that allows for accommodation of the tyrosine mutation at the site (Figure 6h). The neutralization of N501Y+D614G PSV suggests that residue 501 is not critical in the binding site of S‐E6 since no significant change in the neutralization potency is observed between wild‐type and the mutant virus (Figure 5e,f).

### 2.8 SHM Residues Form Specific Interactions with the RBD

Most RBD‐targeting NAbs isolated from COVID‐19 patients have minimal SHM,^[^ ^3^, ^6^, ^31^ ^]^ although some antibodies expressed from memory B cells several months after infection have increased SHM.^[^ ^32^ ^]^ The antibodies derived from the combinatorial antibody library in this study are highly mutated. S‐B8 and S‐E6 contain 13 and 22 SHM residues, respectively, several of which are in the antibody paratope (Figure 6; Figure S8, Supporting Information), including V_H 31_LSH_33_, V_H 50_IT_51_, V_H 53_GD_54_, V_H_ N_56_, V_H_ D_58_, and V_L_ L94 in S‐B8, and V_H_ N_33_, V_L_ V_39_, V_L 50_FDYR_53_, and _65_TR_66_ in S‐E6 (Figure 6c–g; Figure S8, Supporting Information). In summary, several SHM residues appear to be critical for interaction with SARS‐CoV‐2 RBD. The interaction with SHM residues appears to be mainly with the heavy chain in S‐B8 or the light chain in S‐E6. Despite the antibody libraries being generated 20 years ago, this finding implies the possibility that the eliciting antigen, at least to the heavy chain of S‐B8 or light chain in S‐E6, was structurally very similar to the SARS‐CoV‐2 RBD or there is rare but fortuitous cross‐reactivity with another antigen.

### 2.9 Antibody Autoreactivity

To investigate the origin of the three antibodies, a human epithelial type 2 cell (HEp‐2) autoreactivity assay was performed. Neither S‐D4 nor S‐E6 showed a positive signal in the assay, suggesting that they are not derived from an auto‐immune response, indicative of high specificity to S‐RBD (Figure S11a,b, Supporting Information), whereas S‐B8 displayed weak to moderate autoreactivity (Figure S11c, Supporting Information). We further generated an S‐B8 putative germline antibody by mutating back all of the SHMs in the S‐B8 heavy chain to the naïve IGHV3‐66 sequence. The mutated antibody showed greater autoreactivity than S‐B8 (Figure S11d, Supporting Information) and no S‐RBD binding up to 12.5 nm (Figure S12, Supporting Information). Positive and negative controls are shown in Figure S11e,f, Supporting Information.

### 2.10 Antibody‐Dependent Enhancement Activity Assessment of the Three Antibodies

Antibody‐dependent enhancement (ADE) occurs through two distinct mechanisms during viral infections, one via enhanced infection mediated by Fc*γ*RIIa expressed on monocytes and macrophages, and the other via enhanced immune activation caused by excessive Fc‐mediated effector functions and immune complex formation.^[^ ^33^ ^]^ In our antibody constructs, we adopted an engineered IgG4e1(S228P) format to reduce the affinity to Fc*γ* receptors (Fc*γ*Rs). The ADE effects of the three antibodies were assessed in three cell lines expressing different levels of Fc*γ*R using a method recently reported.^[^ ^34^ ^]^ Our qPCR results revealed high‐level Fc*γ*RIa and IIa, high‐level Fc*γ*RIIa, and low‐level to no Fc*γ*RIa, IIa, IIb and IIIa for THP‐1 (a human leukemia monocytic cell line), K562 (a human erythroleukemic cell line), and Raji (a human B lymphoblastoid cell line), respectively (Figure S13a, Supporting Information). Treatment of Raji, K562, and THP‐1 cells with a mixture of SARS‐CoV‐2 PSV with different concentrations of S‐B8, S‐D4, and S‐E6 showed no apparent ADE effects (Figure S13b–d, Supporting Information).

### 2.11 Implications for Origin and Utility of the Combinatorial Antibodies

For over a century, serology has been used to document the origin and presence of infectious agents in patients. Classically, serology depends on the actual presence of specific antibody proteins in the blood. Their target is thought to be the infectious agent, and their presence indicates a relatively recent exposure. By contrast, antibody libraries are nucleic acid based, and include genetic material from memory cells. As such, they provide a record of all of the antibodies that an individual has made, irrespective of whether they are currently being produced. This “fossil record” enabled us to discover SARS‐CoV‐2 NAbs induced either by previous infection or from other immune responses. Furthermore, combinatorial antibody libraries typically yield more diverse antibodies with the desired specificity. During the COVID‐19 pandemic, combinatorial phage libraries as a powerful antibody discovery approach have been used for screening SARS‐CoV‐2 antibodies. Several recent publications reporting NAbs targeting the RBD of SARS‐CoV‐2 spike protein using antibody libraries generated from either naïve or infected human B cells or from a synthetic library.^[^ ^12b–d^ ^]^ Here, we isolated potent and specific antibodies targeting the RBS of SARS‐CoV‐2 spike protein. Of note, these antibodies have no or low autoreactivity in the tests that we conducted and are not reactive with other HCoV spike proteins (Figure 2; Figure S11, Supporting Information). The presence of many somatic mutations in the antibodies isolated from a naïve phage library that are involved in specific interactions with SARS‐CoV‐2 RBD indicates a sustained drive of the immune response to continued presence of a foreign antigen, as occurs during virus replication. Thus, the modern serology detailed here suggests that one of the individuals from whom the library was generated could have been exposed to an antigen with similar surface structure or features to the RBD of SARS‐CoV‐2.

Although the antibody library used here was established in 1999 before the SARS and COVID‐19 pandemics,^[^ ^15^ ^]^ three potent NAbs were discovered in this library. Wec et al. identified several S‐RBD‐directed antibodies that potently cross‐neutralize SARS‐CoV (IC_50_: 0.004–0.06 µg mL^−1^ to PSV) and SARS‐CoV‐2 (IC_50_: 0.05–1.4 µg mL^−1^ to PSV) from memory B cells of a SARS‐CoV donor.^[^ ^4a^ ^]^ They also found over 80% of the low affinity SARS and SARS‐2 cross‐reactive antibodies reacted with one or more of the HCoV spike proteins, such as HCoV‐NL63, HCoV‐229E, HCoV‐OC43, etc., indicating SARS‐CoV infection may have boosted a pre‐existing memory B cell response induced by circulating HCoVs.^[^ ^4a^ ^]^ Several recent publications have also reported cross‐reactive and cross‐NAbs isolated from pre‐pandemic sera targeting highly conserved regions in the S2 domain of human *β*‐coronavirus spike proteins.^[^ ^35^ ^]^ While there is no significant sequence similarity between the RBS of SARS‐CoV‐2 spike protein and these non‐ACE2 targeting human *β*‐coronaviruses, we are not aware of any publication reporting cross‐reactive antibodies targeting the RBS of SARS‐CoV‐2 spike protein. Interestingly, the three antibodies, S‐B8, S‐E6, and S‐D4, identified in this study do not cross‐react with the SARS‐CoV spike protein, which is likely due to differences in the epitope (≈70% difference) in the RBS between SARS‐CoV and SAR‐CoV‐2 targeted by these antibodies (Figure S10, Supporting Information). In addition, none of the three antibodies bind to other five HCoVs (Figure 2c–g). Moreover, autoreactivity assay in a HEp‐2 cell ruled out that S‐E6 and S‐D4 originate from autoimmune responses, whereas S‐B8 showed weak to moderate autoreactivity (Figure S11a–c, Supporting Information), which was increased in the S‐B8 putative germline antibody (Figure S11d, Supporting Information).

Our structural studies on S‐E6 and S‐B8 revealed several striking features of these combinatorial antibodies. The primary immune response to viral infection is followed by a secondary response that generates functionally better antibodies, where the binding energy can be refined by SHM.^[^ ^6^, ^7^, ^14^, ^31^, ^36^ ^]^ The secondary immune response is for later encounter of the same antigen and is the basis of vaccination. In cases of pandemics, such as SARS‐CoV‐2, avian influenza, or Ebola virus, if the infection is not dealt with by the immune system in the first few days, the patient has a high probability of dying, and as a consequence, the immune system will not have enough time to refine the immune response.^[^ ^37^ ^]^ Consistently, NAbs isolated from SARS‐CoV‐2 convalescent patients contain only a few amino‐acid mutations that may be a result of weak B cell stimulation due to rapid viral clearance.^[^ ^3b^ ^]^ NAbs isolated from convalescent patients shortly after infection may then possibly not be fully refined (matured).^[^ ^31^ ^]^ However, a recent study has shown higher levels of SHM several months after infection in some COVID‐19 patients.^[^ ^32^ ^]^ In comparison, S‐B8 and S‐E6 exhibited higher levels of SHM, many of which are involved in specific interactions with SARS‐CoV‐2 RBD. Nine of 13 SHM residues in S‐B8 and eight of 22 in S‐E6 are located in the antibody–antigen interface (Figure S8, Supporting Information). While some of these SHM residues only use their peptide backbone, others rely on specific side chains for S‐RBD binding (Figure 6c–e,g). Interestingly, SHM in CDRH1 of S‐E6 generates a _33_NY_34_ sequence that is similar to the _32_NY_33_ motif in IGHV3‐53/3‐66 antibodies, which are the most frequent germlines used in targeting the S‐RBD, indicative that the combinatorial antibody library and the maturation process can yield alternate antibody solutions (Figure S8, Supporting Information). However, it is unclear how these SHM residues could have been raised specifically to the SARS‐CoV‐2 RBD, since the library was generated long before the SARS‐CoV‐2 pandemic. Of note, the heavy and light chains are randomly paired during our selection experiment. However, S‐E6 is a light‐chain dominant antibody and most of the SHM residues in the heavy chain are not involved in interaction with SARS‐CoV‐2 RBD (Figure S8, Supporting Information). Thus, these findings raise fascinating questions about the original antigen(s) that elicited S‐B8 and S‐E6, at least to the heavy or light chains that dominate binding to SARS‐CoV‐2 RBD.

The unnaturally paired antibodies in a combinatorial scFv antibody library also allow one to identify other alternative solutions with high binding affinity and efficacy.^[^ ^14a^ ^]^ Relative rare germline antibodies can be enriched during the iterative affinity panning, as for example for S‐E6, which is an IGHV4‐31 antibody less frequently seen in NAbs from convalescent patients in comparison to the database collected as of July 24, 2021 (Figure S14, Supporting Information).

A general question posed by these studies is whether therapies based on the vast number of starting antibodies in combinatorial libraries could be more powerful than the antibodies generated in vivo during the limited time available for affinity‐based antibody evolution and selection in the setting of an acute and potentially lethal infection. These issues also have direct relevance in the clinical setting, where a major concern in antibody therapy is the high mutation rate of viral spike proteins, which can render prior highly specific antibodies unable to recognize or neutralize mutant viruses, as currently observed in alpha,^[^ ^19^ ^]^ beta,^[^ ^38^ ^]^ gamma,^[^ ^21^ ^]^ and delta^[^ ^39^ ^]^ variants. Mixtures of antibodies targeting distinct epitopes can be used to overcome such immune escape.^[^ ^40^ ^]^

## 3 Conclusion

In the present study, antibodies identified from combinatorial libraries with high SHM and rare germline derivation, in combination with antibodies from convalescent patients, or with convalescent plasma, could provide yet another therapeutic option and a potential antidote to immune escape. Knowledge of the evolution of immune response in terms of the interactions of NAbs and their binding epitopes on SARS‐CoV‐2 also provides a blueprint for next‐generation vaccine design. Since a complete antibody repertoire of an individual is the starting reservoir of all possibilities, one can also learn much more about the origins and evolution of an immune response against viral challenge when it is studied.^[^ ^14a^ ^]^ The observation of highly potent NAbs from a library of “healthy” donors before the COVID‐19 pandemic could also indicate possible prior exposure of a donor(s) to a similar coronavirus 20 years ago or to an antigenic surface on a protein that has features resembling the SARS‐CoV‐2 RBD. However, whether these antibodies are a consequence of background immunity remains to be elucidated. Due to random coupling of V_H_ and V_L_ sequences in the original combinatorial antibody library, the antibodies isolated here do not fully represent the natural selection process of the human B cell repertoire. However, the genes encoding either heavy chain or light chain are of natural origin. The heavy chain of S‐B8 and light chain of S‐E6 that dominate binding to SARS‐CoV‐2 RBD have many somatically mutated residues (12 for S‐B8 heavy chain and 15 for S‐E6 light chain) involved in key interactions with the RBD. Notwithstanding, we cannot rule out potential implications of such findings from libraries made decades ago concerning the origin of the viruses currently circulating.

## 4 Experimental Section

### Cell Culture

The Vero cell line (ATCC CCL‐81) was maintained in a DMEM/F‐12k media (Gibco, #C11330500CP) containing 10% v/v FBS (Gibco, #1600074). The FreeStyle 293‐F (HEK 293F, ThermoFisher Scientific, #R79007) cell line was cultured in a Freestyle 293 expression media (ThermoFisher Scientific, #12338026). For establishing the HEK293T/*h*ACE2 stable cell line, HEK293T cells (ATCC ACS‐4500) were transiently transfected with *h*ACE2 fusion BFP encoding PB510 plasmid using PiggyBac Transposon System (System Biosciences, PB210PA‐1), followed by addition of 2 µg mL^−1^ puromycin 6 h post‐transfection. The resulting cells were kept in puromycin‐containing media for an extra 2 days. Positive cells with BFP expression were sorted by a flow cytometry instrument (BD FACS Aria III). The sorted cells with overexpressed *h*ACE2 were expanded and cultured in a DMEM media (Gibco, #10566016) supplemented with 10% FBS v/v and 10 µg mL^−1^ puromycin.

### Expression and Purification of Recombinant SARS‐CoV‐2 Spike RBD, Human ACE2, and Antibodies

The DNA sequences of codon‐optimized SARS‐CoV‐2 Spike RBD (S‐RBD) and human ACE2 extracellular domain (*h*ACE2‐ECD) were cloned into a *p*Fuse‐Fc expression vector (Invivogen). A thrombin cleavage sequence was inserted between the RBD and Fc to generate a cleavable *h*Fc tag for future studies. The SARS‐CoV‐2 RBD‐*h*Fc and *h*ACE2‐ECD‐*m*Fc proteins were heterologously expressed in HEK293F cells by transient transfection and cultured for 4 days, then purified by Mabselect columns (Cytiva, #17‐5199‐01). Briefly, cell media with secreted Fc tagged recombinant proteins, S‐RBD‐*h*Fc and *h*ACE2‐ECD‐*m*Fc, were loaded onto a Mabselect column that was pre‐washed and equilibrated with a PBS buffer (150 mm NaCl, 20 mm sodium phosphate, pH 7.2), and eluted using a pH 3.4 citrate acid buffer. DNA sequences for the variable regions of the combinatorial antibodies were cloned into a full‐length human IgG4 mutant construct (S228P) and expressed in HEK293F cells for 4 days and further purified by Mabselect chromatography. Purified recombinant proteins and antibodies were buffer‐exchanged into a PBS buffer using centrifugal concentrators.

### Function‐Guided Phage Panning

SARS‐CoV‐2 RBD specific scFv antibodies were selected from a combinatorial human monoclonal scFv antibody phage library (10^11^ members) after two rounds of affinity enrichment against the biotinylated S‐RBD protein immobilized on the SA‐coated magnetic beads (Pierce, #21925), followed by a third round of competitive panning versus *h*ACE2‐ECD protein. Briefly, phagemid (displaying the antibody library) binding to the antigen (S‐RBD) was enriched at each cycle and eluted with Glycine‐HCl (pH 2.2) in the first two rounds of screening. XL1‐Blue cells were used to express and amplify the output phagemids for the next round of panning. To determine *h*ACE2 competitive antibodies, a kinetic competitive panning method was adopted in the third‐round panning. Instead of the conventional pH 2.2 buffer, an elution buffer containing a saturated concentration of *h*ACE2‐ECD protein (200 nm; for S‐RBD and *h*ACE2‐ECD binding, EC_80_ = 80 nm) was used to elute the phagemids twice. The method used for output titering was as described.^[^ ^41^ ^]^ In brief, 200 µL of eluted phages were first diluted into 2 mL of super‐broth (SB) medium, then 1 µL of the above elute was further serial diluted 10^−2^, 10^−4^, and 10^−6^ times in a final volume of 100 µL SB. The 100 µL of the diluted elute were plated on Luria–Bertani agar plates and incubated overnight. The colonies on each plate were counted and the output size calculated by multiplying the number of colonies by the dilution factor. After three iterations, 96 colonies were selected and analyzed by phage ELISA as described.^[^ ^41^ ^]^ All positive clones were sequenced using Sanger sequencing. The DNA and protein sequences of CDR3 domains were analyzed using the international IMGT information platform (http://www.imgt.org/).

### Phage ELISA

Avidin (Pierce, #21121) was diluted to a final concentration of 2 ng µL^−1^ in a PBS buffer (Sigma, #C3041). The resulting avidin solution was used to coat the 96‐half well plates (25 µL per well) at 4 °C overnight. The coated plates were washed once with the PBS buffer (150 µL per well) followed by the addition and incubation of 25 µL biotinylated SARS‐CoV‐2 RBD‐*h*Fc solution (2 ng µL^−1^) in each well at room temperature for 1 h. The PBST (PBS containing 0.05% Tween‐20) buffer alone and the *h*Fc solution (2 ng µL^−1^) were used as the background and negative controls, respectively. After removal of the incubation solution, the resulting plates were rinsed once using the PBST buffer and incubated with a blocking solution containing 5% milk v/v in PBST (150 µL per well) at 37 °C for 1 h. After blocking and PBST washing (once), 50 µL of phagemid‐containing XL1‐Blue culture medium supernatants (by centrifuging the third‐round panning output XL1‐Blue cells at 3000 g, 15 min) mixed with 10 µL 5% milk v/v in PBST was added to each well and incubated at 37 °C for 1 h. The resulting plates were rinsed eight times using PBST before subjecting to horseradish peroxidase (HRP) detection. A solution containing the secondary antibody, anti‐M13 bacteriophage antibody conjugated with HRP (dilution factor 1:5000; Sino Biological, #11973‐MM05T‐H), was added into the above plates (150 µL per well) and incubated at 37 °C for 1 h. Plates were then washed eight times with PBST followed by the addition of 50 µL ABTS solution (Roche, #11684302001) into each well. After ≈10 min incubation at room temperature, the absorbance change at 405 nm in each well was measured on a microplate reader (Enspire, PerkinElmer).

### Competitive ELISA

Competition between the selected antibodies and *h*ACE2 for binding to the SARS‐CoV‐2 spike protein RBD was measured. The recombinant *h*ACE2‐ECD was coated in PBS buffer at 2 ng µL^−1^, 100 µL per well at 4 °C overnight, washed with PBS once, then blocked with 3% BSA in PBS. Biotinylated S‐RBD (*h*Fc tag removed by thrombin digestion) at a final concentration of 50 nm was incubated with twofold serial diluted S‐B8, S‐D4, and S‐E6 antibodies (from 1 to 133 nm) at 4 °C for 30 min, in which an IgG4e1 isotype antibody was used as the negative control. The S‐RBD and antibody mixture was then added to the *h*ACE2‐ECD coated plates and incubated at room temperature for 1 h, followed by 4 washes with PBST. The *h*ACE2‐ECD bound S‐RBD in the plate was detected using a SA‐HRP conjugated protein.

### Affinity Determination by Biolayer Interferometry

Binding affinities of IgG S‐D4 with SARS‐CoV‐2 wild‐type or mutant S‐RBD were performed by BLI on an Octet RED96 (Molecular Devices LLC, San Jose, CA, USA) using AR2G biosensors. The SARS‐CoV‐2 RBD fused *h*Fc was first digested by thrombin to remove the Fc tag. The resulting S‐RBD diluted in a PBS solution containing 0.02% Tween‐20 and 0.05% BSA (PBST‐B) (10 µg mL^−1^) was loaded to the AR2G biosensor by amine coupling. The AR2G‐S‐RBD sensors were dipped into a PBST‐B for 60 sec to establish a baseline, and then incubated with twofold serial diluted antibody solutions to record the progressive curves of association. Finally, sensors were incubated in a PBST‐B buffer to record the progressive curves of dissociation. For IgG S‐B8 and S‐E6 detections, S‐RBD was first biotinylated before loading to a SA sensor, the remaining procedure was same to that of S‐D4. Sensor regeneration was performed by dipping the used sensors into a pH 3.4 citrate acid buffer and equilibrated in a PBST‐B buffer. For further kinetics analysis, S‐B8, S‐E6, and S‐D4 Fabs were immobilized on the Fab2G biosensor and titrated with serially diluted SARS‐CoV‐2 RBD in kinetics buffer (1× PBS, pH 7.4, 0.002% Tween‐20, 0.01% BSA) for 180 sec followed by disassociation in kinetics buffer for 180 sec. All experimental data were analyzed by ForteBio Data Analysis software with a 1:2 binding model (bivalent analyte) for IgG and a 1:1 binding model for Fab data with *R*_max_, *k*_on_, and *k*_off_ fitted globally.

### Interaction of Antibodies with Cell Surface Expressed Spike by FACS

In a flow‐cytometry binding experiment, the spike protein of full‐length SARS‐CoV‐2, SARS‐CoV, or other HCoVs, which was conjugated with P2A‐EGFP, was transiently transfected into HEK293T cells. After 24 h cultivation, cells were collected and re‐suspended in an ice‐cold FACS buffer (PBS, 0.05% BSA, and 2 mm EDTA). The spike protein expressing cells (50 000 cells per tube) were then incubated with different anti‐S‐RBD antibodies for 20 min at 4 °C, and washed with 1 mL ice‐cold FACS buffer, spun, and re‐suspended in a 100 µL ice‐cold FACS buffer containing the Alexa555 conjugated secondary antibody that recognizes *h*Fc (1:800 v/v dilution, Life technology, # A21433). After incubating at 4 °C for 15 min, the cells were washed twice and re‐suspended in a FACS buffer, and then sorted and analyzed on a flow cytometer (CytoFLEX S, Beckman Coulter) to determine relative binding level by the antibodies to the cell overexpressing wild‐type spikes. Mean fluorescence intensities of Alexa555 in EGFP‐positive cells were recorded and analyzed to evaluate antibody binding.

### Size‐Exclusion‐High‐Performance Liquid Chromatography

Twenty µL of 0.5 µg µL^−1^ purified S‐RBD antibodies were applied to an Agilent Bio SEC‐5, 500 A HPLC system. The mobile phase used PBS buffer (pH 7.2) running at a flow rate of 0.35 mL min^−1^. Absorbance was analyzed by retention time to determine the percentage of aggregation, monomer, and degradant compositions.

### Cell–Cell Fusion Assay

The cell–cell fusion assay was established according to a previous report with minor modifications.^[^ ^42^ ^]^ Briefly, *h*ACE2 positive Vero cells (cells with endogenous *h*ACE2 were sorted by FACS) were used as target cells. HEK293F cells that were transiently transfected with either SARS‐CoV‐2 spike‐P2A‐EGFP or SARS spike‐P2A‐EGFP were set as effector cells. The target Vero cells were first seeded into 24‐well plates at a density of 1 × 10^5^ per well and cultivated at 37 °C for 4 h, followed by addition of effector cells, HEK293F/SARS spike‐EGFP or HEK293F/SARS‐CoV‐2 spike‐EGFP, at a ratio of 2:1, respectively. The co‐cultures of cells were cultivated in a DMEM medium with 10% FBS and treated with or without anti‐SARS‐CoV‐2 spike antibodies at indicated concentrations. The recombinant SARS‐CoV‐2 RBD was used as a positive control. After cultivating at 37 °C for 6 h, the rates of cell–cell fusion were evaluated using a fluorescence microscope (EVOS M5000, Life Technologies). Five fields for microscopic analysis were randomly selected in each treated group, the numbers of fused and unfused EGFP positive cells were counted.

### Preparation of Pseudovirus

HEK293T cells were co‐transfected with both NL4‐3 mCherry Luciferase plasmid (Addgene, #44965) and pcDNA3.1 wild‐type or mutant SARS‐CoV‐2 spikeΔ19 plasmid (encoding SARS‐CoV‐2 spike protein, with 19 AA truncated in C terminal) using Lipofectamine 3000 (Invitrogen, #L3000‐015) following the manufacturer's instruction. Pseudotyped particles were readily released into the supernatant. The supernatants containing SARS‐CoV‐2 PSV were harvested at 48 h post‐transfection, filtered (0.45 µm pore size, Sartorius, #16533‐K), and mixed with the Lenti‐X Concentrator (Takara, #631231) overnight at 4 °C. The mixture was then centrifuged at 1500 g for 45 min at 4 °C. The cell pellets were collected and re‐suspended in a DMEM medium and stored at −80 °C until use.

### Pseudovirus‐Based Neutralization Assay

To detect the neutralization ability of selected antibodies against infection of coronavirus PSV, HEK293T/*h*ACE2 cells were first seeded into 96‐well, white‐bottom plates at a density of 1 × 10^4^ per well and cultivated overnight. The PSV was pre‐incubated with an equal volume of different concentrations of selected antibodies (dilution factor: 3.16, from 200 nm to 200 fM for S‐B8 and S‐D4, 200 nm to 6.3 fm for S‐E6 in WT PSV detection, and others are as indicated) in DMEM at 37 °C for 30 min. DMEM with or without PSV in the absence of antibodies were set as controls. After incubation, the PSV mixture was transferred to the culture plates containing HEK293T/*h*ACE2 cells. The DMEM media containing PSV and antibodies were replaced with fresh media after 16 h treatment, cells were incubated for an additional 48 h. PSV infection efficacy was evaluated by luciferase activity using Bright‐Lumi Firefly Luciferase Reporter Gene Assay Kit (Beyotime, #RG015M). Fifty microliter of luciferase substrate was added to each well, and the relative luminescence unit values were measured on an Envision plate reader (PerkinElmer, Ensight). The antibody concentration was first transformed into Log(X), and the least squares fit (Y = Bottom+(Top‐Bottom)/(1+10^((LogIC50‐X)*HillSlope))) was then used for non‐linear regression analysis in GraphPad Prism 8.3.

### Authentic SARS‐CoV‐2 Virus Neutralization Assay

The study was performed in the CL3 Facility of the University of Oxford operating under license from the HSE, on the basis of an agreed Code of Practice, Risk Assessments (under ACDP) and Standard Operating Procedures. The microneutralization protocol is similar to that described in D. T. Skelly, et al., 2021.^[^ ^43^ ^]^ In brief, this rapid, high‐throughput assay determines the concentration of antibody that produces a 50% reduction in infectious focus‐forming units of different authentic SARS‐CoV‐2 strains in Vero cells, as follows. Quadruplicate, 0.5log_10_ serial dilutions of antibody (11 steps from 100 nm to 1 pM) were pre‐incubated with a fixed dose of SARS‐CoV‐2 (Victoria 01/2020 isolate) before incubation with Vero cells. A 1.5% carboxymethyl cellulose‐containing overlay was used to prevent satellite focus formation. Twenty hours post‐infection, the monolayers were fixed with 4% paraformaldehyde, permeabilized with 2% Triton X‐100 and stained for N antigen using mAb EY 2A.^[^ ^44^ ^]^ After development with a peroxidase‐conjugated antibody and True Blue peroxidase substrate, infectious foci were enumerated by ELISPOT reader. Data were analyzed using four‐parameter logistic regression (Hill equation) in GraphPad Prism 8.3.

### Autoreactivity Assay

The autoreactivity assay was performed using a HEp‐2 anti‐nuclear antibodies kit (Medical and Biological Laboratories Co., Ltd, #4220‐12CN) according to the manufacturer's instructions. Briefly, 35 µL of 0.1 mg mL^−1^ antibodies were loaded to the wells in a slide pre‐seeded with fixed and permeabilized HEp‐2 cells and incubated for 20 min at room temperature. Positive serum from autoimmune patients and negative serum from healthy donors provided by the kit were used as controls. After washing twice (5 min each), the FITC‐conjugated secondary anti‐human antibody was incubated with the cells for 20 min at room temperature. The slide was then washed and mounted with a coverslip before observation on a fluorescent microscope (ZEISS, Axio Observer A1) with a 20× objective.

### Protein Production and Structure Determination

The coding sequence for RBD (residues 319–541) of the SARS‐CoV‐2 spike (S) protein was synthesized and cloned into a customized pFastBac vector,^[^ ^45^ ^]^ which was designed to fuse an N‐terminal gp67 signal peptide and C‐terminal His_6_‐tag to the target protein. To express the RBD protein, a recombinant bacmid DNA was generated from the sequencing‐confirmed pFastBac construct using the Bac‐to‐Bac system (Life Technologies). Baculovirus was generated by transfecting purified bacmid DNA into Sf9 cells using FuGENE HD (Promega), and subsequently used to infect suspension cultures of High Five cells (Life Technologies) at a multiplicity of infection of 5 to 10. Infected High Five cells were incubated at 28 °C with shaking at 110 rpm for 72 h for protein expression. RBD protein that was secreted into the supernatant was harvested and then concentrated with a 10 kDa MW cutoff Centramate cassette (Pall Corporation). The RBD protein was purified by affinity chromatography using Ni‐NTA resin (QIAGEN), followed by size exclusion chromatography on a HiLoad Superdex 200 pg column (GE Healthcare), and buffer exchanged into 20 mm Tris‐HCl pH 7.4 and 150 mm NaCl using the same protocol as previously described.^[^ ^46^ ^]^ Fabs were expressed in ExpiCHO cells and purified using CaptureSelect CH1‐XL resin (ThermoFisher) and followed by size exclusion chromatography. The Fab+RBD complexes were formed by mixing the two components in an equimolar ratio and incubating overnight at 4 °C before setting‐up crystal trials. The Fab/RBD complexes were screened for crystallization using 384 conditions of the JCSG Core Suite (QIAGEN) on the robotic CrystalMation system (Rigaku) at The Scripps Research Institute. Crystals appeared in the first week, were harvested during the second week, and then flash‐cooled in liquid nitrogen for X‐ray diffraction experiments. Diffraction data were collected at cryogenic temperature (100 K) at beamline 23‐ID‐B of the Advanced Photon Source (APS) at Argonne National Laboratory with a beam wavelength of 1.033 Å and processed with HKL2000.^[^ ^20^ ^]^ Diffraction data were collected from crystals grown in conditions: 20% PEG 3350, 0.2 m sodium sulfate, and pH 6.6 for the S‐B8+RBD complex; and 20% isopropanol, 20% PEG 4000, 0.1 m citrate, and pH 5.6 for the S‐E6+RBD complex. The X‐ray structures were solved by molecular replacement (MR) using PHASER^[^ ^47^ ^]^ with MR models for the RBD and Fab from PDB 7JMW.^[^ ^18a^ ^]^ Iterative model building and refinement were carried out in COOT^[^ ^48^ ^]^ and PHENIX,^[^ ^49^ ^]^ respectively. Epitope and paratope residues, as well as their interactions, were identified by using PISA program^[^ ^50^ ^]^ with BSA (>0 Å^2^) as the criterion.

### Statistical Analysis

The results were expressed as means ± standard deviation (SD) unless otherwise indicated, and the sample numbers are indicated in figure legend. Data analysis was performed by using Origin Pro 2019 statistical software or GraphPad Prism software. Significance was assumed at a *p* value < 0.05 by using Student's *t*‐test in GraphPad Prism software.

## Conflict of Interest

The authors declare no conflict of interest.

## Author Contributions

M.Q., P.X.M., Y.L., and H.L. contributed equally to this work. R.A.L., G.Y., H.W., W.J., R.A.D, and I.A.W. conceived the project. M.Q., P.X.M., H.L., P.D.T., and X.J.S. contributed to project design and extensive discussions. M.Q., P.X.M., Y.L., F.L.W, L.L.L., C.Y.M., Q.J., P.D.T., Z.A.L., A.S., T.L., X.W., C.Y.Z., and Y.Z. performed antibody selection, identification, binding, cell–cell fusion, and PSV neutralization work. H.L., M.Y., N.C.W., C.‐C.D.L., and X.Z. performed structural work involving protein production, crystallization, structure determination, and analysis. W.J., A.H., and J.G.‐J. performed the authentic virus neutralization experiments. G.Y., M.Q., P.X.M., H.L., I.A.W., and R.A.L. analyzed data and wrote the manuscript. X.X.H., W.J., and R.A.D. provided manuscript edits and suggestions.

## Supporting information

Supporting Information

## Data Availability

The RCSB protein data bank (PDB) has hold the two accession codes pending for citation updates. We have informed the PDB stuff with the updated citation information. The structures will be released in the next Wednesday with DOIs: 10.2210/pdb7KN3/pdb for 7KN3 and 10.2210/pdb7KN4/pdb for 7KN4.
