# Supplementary material for: Neutralizing Antibodies to SARS‐CoV‐2 Selected from a Human Antibody Library Constructed Decades Ago
Source: Adv Sci (Weinh). 2021 Oct 29;9(1):2102181. doi: 10.1002/advs.202102181 (PMC8646600; doi:10.1002/advs.202102181)
Supplement: Supplementary file 1 — Supporting Information [file ADVS-9-2102181-s001.pdf]

ADVANCED SCIENCE
Open Access

Supporting Information

for *Adv. Sci.*, DOI: 10.1002/advs.202102181

Neutralizing Antibodies to SARS-CoV-2 Selected from a Human Antibody Library Constructed Decades Ago

Min Qiang, Peixiang Ma, Yu Li, Hejun Liu, Adam Harding, Chenyu Min, Fulian Wang, Lili Liu, Meng Yuan, Qun Ji, Pingdong Tao, Xiaojie Shi, Zhean Li, Teng Li, Xian Wang, Yu Zhang, Nicholas C. Wu, Chang-Chun D. Lee, Xueyong Zhu, Javier Gilbert-Jaramillo, Chuyue Zhang, Abhishek Saxena, Xingxu Huang, Hou Wang, William James, Raymond A. Dwek, Ian A. Wilson, Guang Yang*, and Richard A. Lerner**

Supporting Information for:

Neutralizing Antibodies to SARS-CoV-2 Selected from a Human Antibody Library Constructed Decades Ago

Min Qiang, Peixiang Ma, Yu Li, Hejun Liu, Adam Harding, Chenyu Min, Fulian Wang, Lili Liu, Meng Yuan, Qun Ji, Pingdong Tao, Xiaojie Shi, Zhean Li, Teng Li, Xian Wang, Yu Zhang, Nicholas C. Wu, Chang-Chun D. Lee, Xueyong Zhu, Javier Gilbert-Jaramillo, Chuyue Zhang, Abhishek Saxena, Xingxu Huang, Hou Wang, William James, Raymond A. Dwek, Ian A. Wilson, Guang Yang*, and Richard A. Lerner**

Dr. M. Qiang, Dr. P. Ma, Y. Li, F. Wang, L. Liu, Q. Ji, Dr. P. Tao, Dr. X. Shi, Dr. Z. Li, T. Li, X. Wang, Chu. Zhang, Dr. A. Saxena, Prof. G. Yang
Shanghai Institute for Advanced Immunochemical Studies
ShanghaiTech University
Shanghai 201210, P.R. China.
Email: yangguang@shanghaitech.edu.cn

Prof. R. Lerner
Department of Chemistry
The Scripps Research Institute
La Jolla, CA 92037, USA.
Email: rlerner@scripps.edu

Dr. H. Liu, Dr. M. Yuan, Dr. N. C. Wu, Dr. C.-C. D. Lee, Dr. X. Zhu, Prof. I. A. Wilson
Department of Integrative Structural and Computational Biology
The Scripps Research Institute
La Jolla, CA 92037, USA.
Email: wilson@scripps.edu

A. Harding, J. Gilbert-Jaramillo, Prof. W. James
Sir William Dunn School of Pathology
University of Oxford, Oxford, OX1 3RE, UK.

C. Min, Prof. G. Yang
Velox Pharmaceuticals
Changzhou 213000, P.R. China.

Prof. R. A. Dwek
Oxford Glycobiology Institute, Department of Biochemistry
South Parks Road, Oxford OX1 3QU, UK.

Y. Li, F. Wang, P. Tao, T. Li, X. Wang, C. Zhang, Dr. Y. Zhang, Prof. X. Huang

School of Life Science and Technology
ShanghaiTech University
Shanghai 201210, P.R. China.

Y. Li, F. Wang, P. Tao, T. Li, X. Wang, C. Zhang
Institute of Biochemistry and Cell Biology
Shanghai Institutes for Biological Sciences, Chinese Academy of Sciences
Shanghai 200031, P.R. China.

Y. Li, F. Wang, P. Tao, T. Li, X. Wang, C. Zhang
University of Chinese Academy of Sciences
Beijing 100049, P.R. China.

Prof. I. A. Wilson
The Skaggs Institute for Chemical Biology
The Scripps Research Institute
La Jolla, CA 92037, USA.

H. Wang,
ShOx Science Limited
Shanghai 200135, P.R. China.

M. Q., P. M., Y. L., and H. L. contributed equally to this work.

*Corresponding author
E-mail: wilson@scripps.edu (I.A.W.), yangguang@shanghaitech.edu.cn (G.Y.), rlerner@scripps.edu (R.A.L.).

This PDF file includes:

Figure S1 to S14

Table S1 to S2

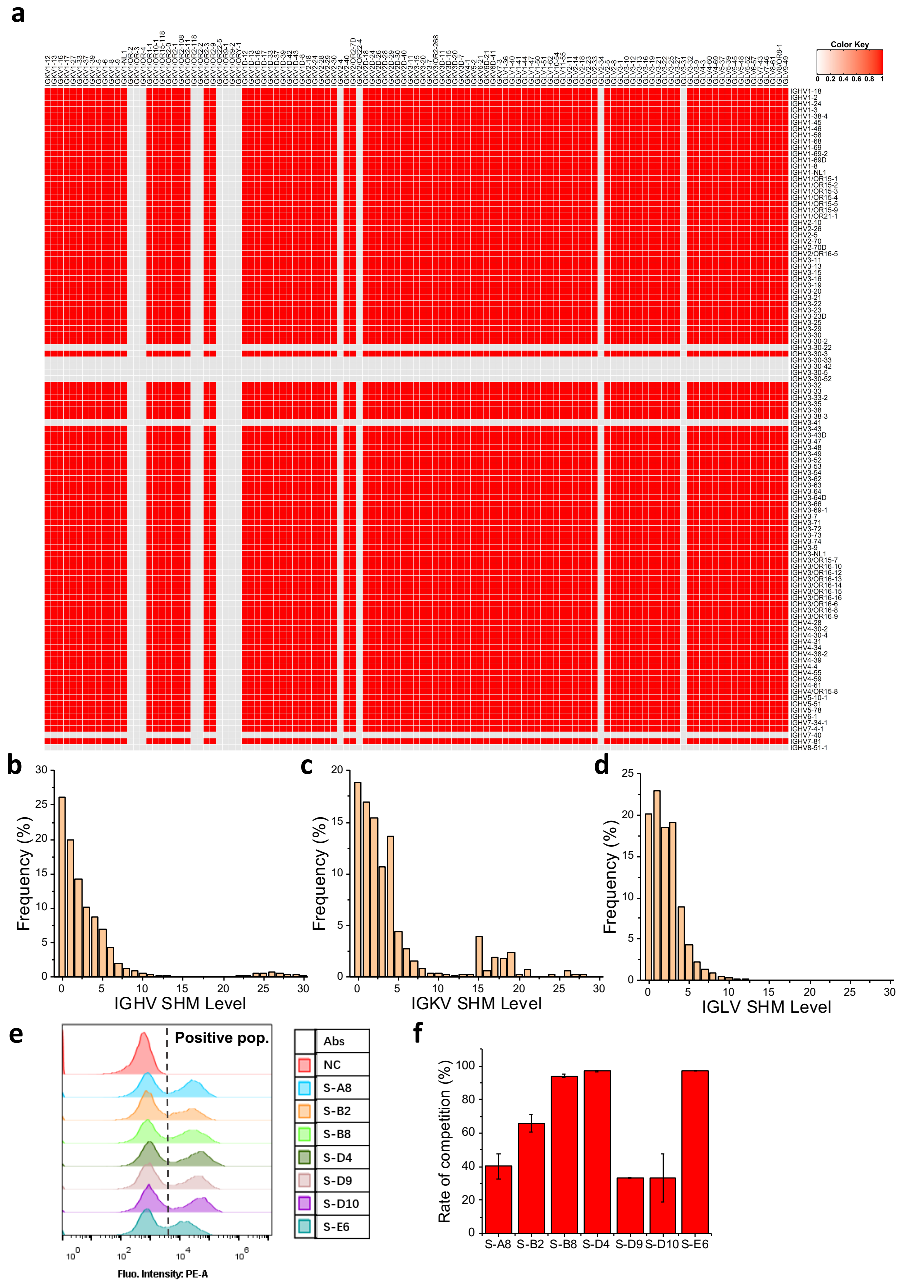

Figure S1. Germline matrix coverage and SHM statistics of the naïve antibody library with screening strategies for final antibody selection. a) The heavy and light chain genes of the naïve antibody library were separately sequenced by next generation sequencing, and the IGHV *vs*. IGLV and IGKV germline matrix coverage hotmaped (red color) as shown and aligned to the full germline distribution derived from IMGT (international ImMunoGeneTics) database (http://www.imgt.org/). b-d) The SHM at the amino-acid level of IGHV, IGKV and IGLV in a

total of 400,000 sequences from the naïve antibody library were calculated and plotted as shown. e) Supernatants of the 22 IgG4e1 antibodies were tested for binding to the SARS-CoV-2 spike protein. HEK293T cells transiently transfected with SARS-CoV-2-P2A-EGFP were incubated with the supernatants containing secreted antibodies, followed by staining with anti-human IgG-Alexa 555 conjugated 2nd antibody for FACS analysis. The positive population was revealed by the appearance of a PE signal, and seven of the initial 22 antibodies were shown to bind to the full-length SARS-CoV-2 spike protein. f) Competitive ELISA was performed for the seven antibodies selected in **e** to assess competition with ACE2. *h*ACE2-ECD was coated onto a 96-well plate, and 10 nM of S-RBD was first incubated with 100 μL of antibody supernatant prior to adding to the plate. The binding signal (OD_{405}) of SARS-CoV-2 RBD was recorded for the competition analysis. The percentage of ACE2 competition with each antibody is shown in a bar chart where the error bar indicates the SD (n=3).

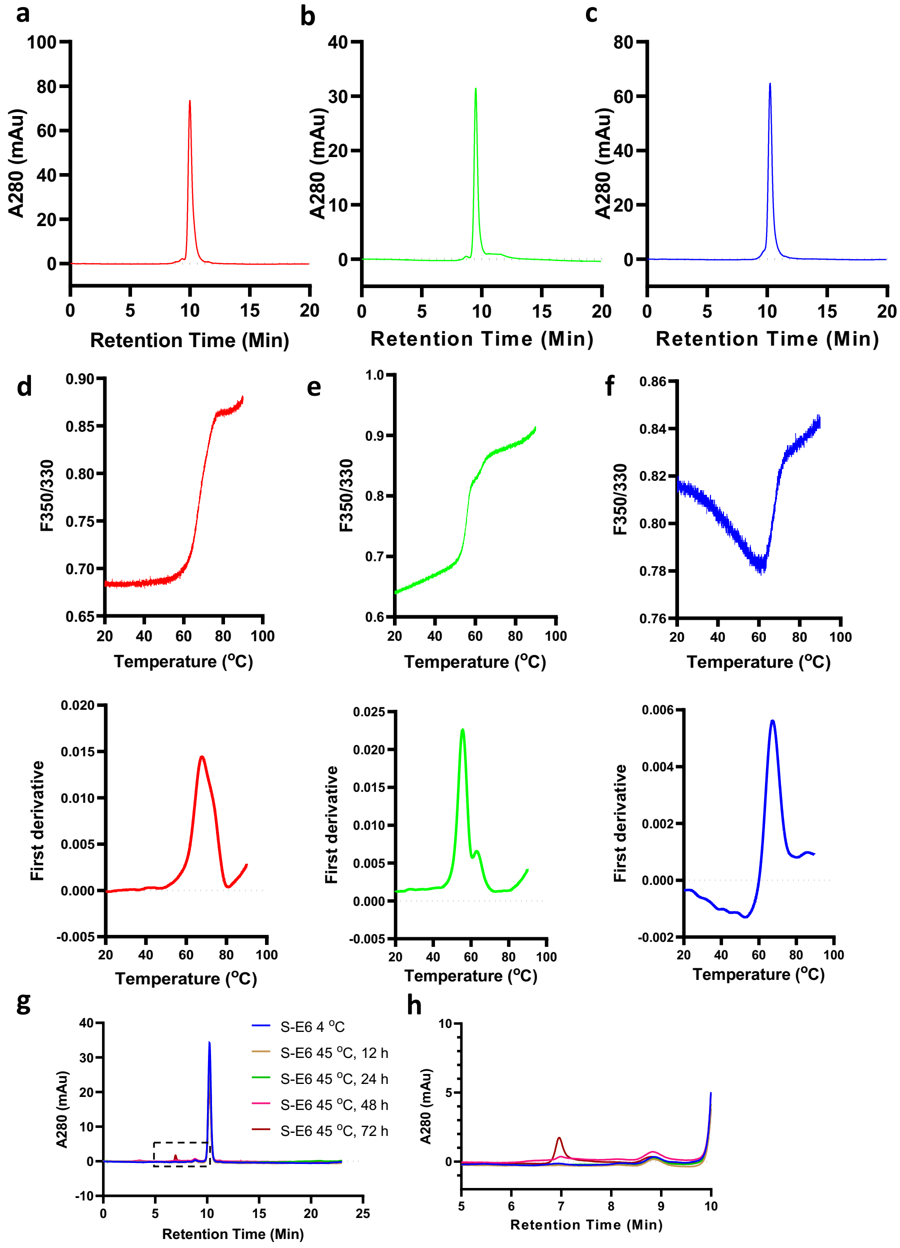

Figure S2. HPLC-SEC and nanoDSF thermostability assay of selected antibodies. 0.5 mg/mL full-length S-B8 (a), S-D4 (b) and S-E6 (c) IgG4e1 proteins were used in the HPLC-SEC characterization. DSF fluorescence ratios (F350/F330) for S-B8 (d), S-D4 (e) and S-E6 (f) were measured as melting curves in upper panel with their corresponding first derivative curves shown in the lower panel. The derivative plot for each antibody identifies the inflection point in the curve as a peak at 67.8 °C, 55.7 °C, and 66.3 °C, respectively. g-h) HPLC-SEC assessment of S-E6 thermal stability. Aliquots of S-E6 IgGe1 protein dissolved in PBS buffer (150 mM NaCl, 20 mM

sodium phosphate, pH 7.2) were incubated at 4 ºC or 45 ºC with indicated time intervals and subjected to analysis in an Agilent Bio SEC-5, 500A HPLC system running with the same PBS buffer at a flow rate of 0.35 mL/min. S-E6 showed high stability under different incubation times (g) despite a small fraction of aggregates appears after incubating for 48 hours at 45 ºC (h). The dashed box in g indicates the zoomed-in view in h.

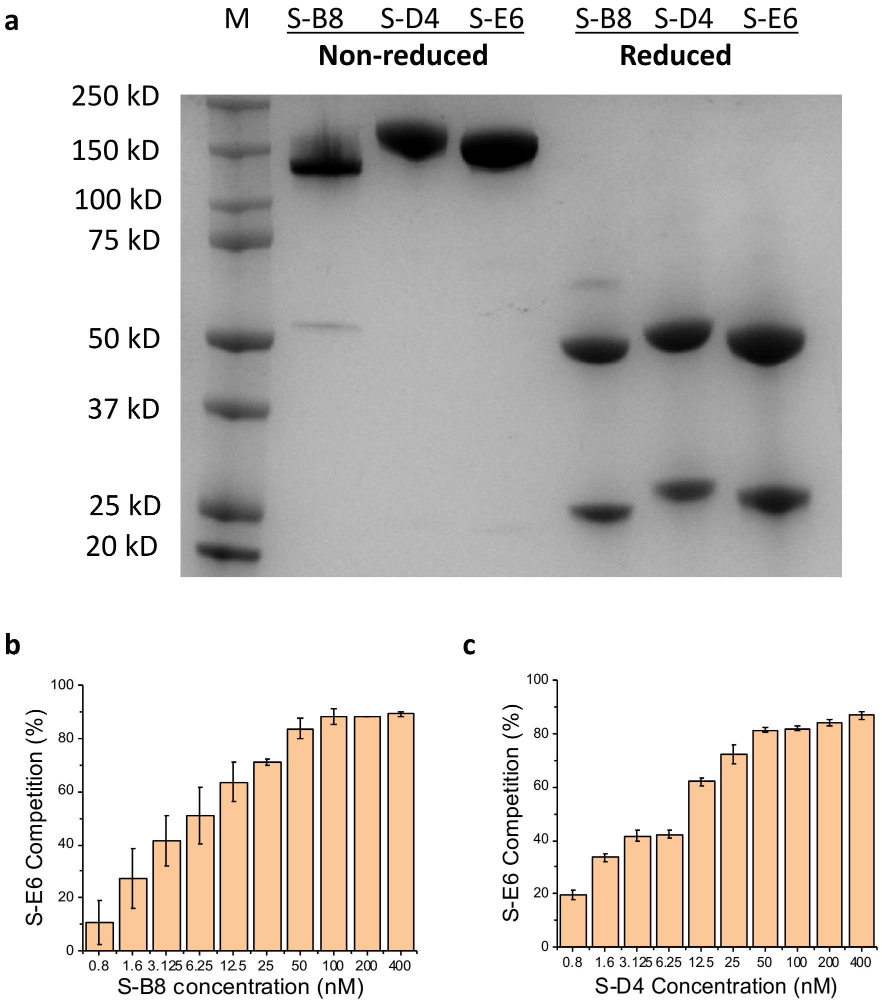

Figure S3. SDS-PAGE analysis of *h*ACE2 competitive antibodies and competition assay between Abs. a) Three *h*ACE2 competitive antibodies in IgG4e1 format were analyzed by SDS-PAGE, in non-reduced and reduced forms. b, c) S-E6 was coated onto 96-well plates and different concentrations of S-B8 (b) or S-D4 (c) were pre-incubated with S-RBD before competitive binding with S-E6. The inhibition of binding of S-E6 to S-RBD by S-B8 and S-D4 as a percentage is shown.

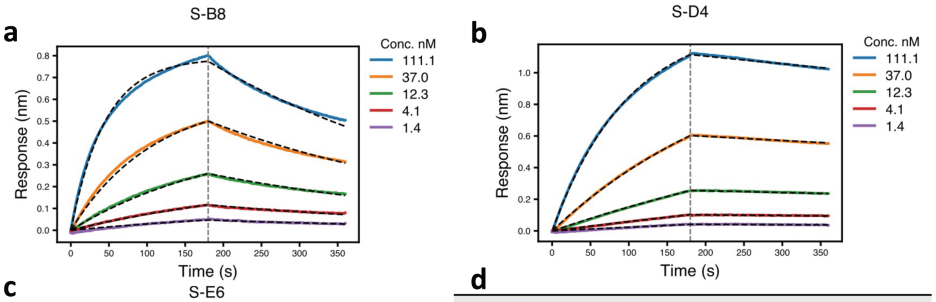

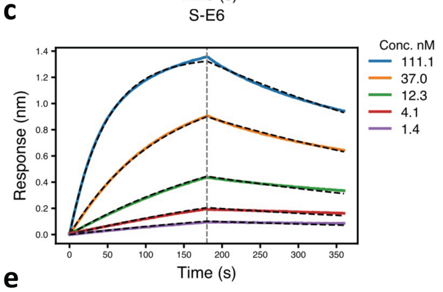

d

Abs	K_{on} (1/Ms)	K_{off} (1/S)	K_D (M)	R^2
S-B8 Fab	1.76×10^5	2.70×10^{-3}	1.54×10^{-8}	0.9986
S-D4 Fab	8.69×10^4	4.50×10^{-4}	5.18×10^{-9}	0.9998
S-E6 Fab	1.72×10^5	1.96×10^{-3}	1.14×10^{-8}	0.9996

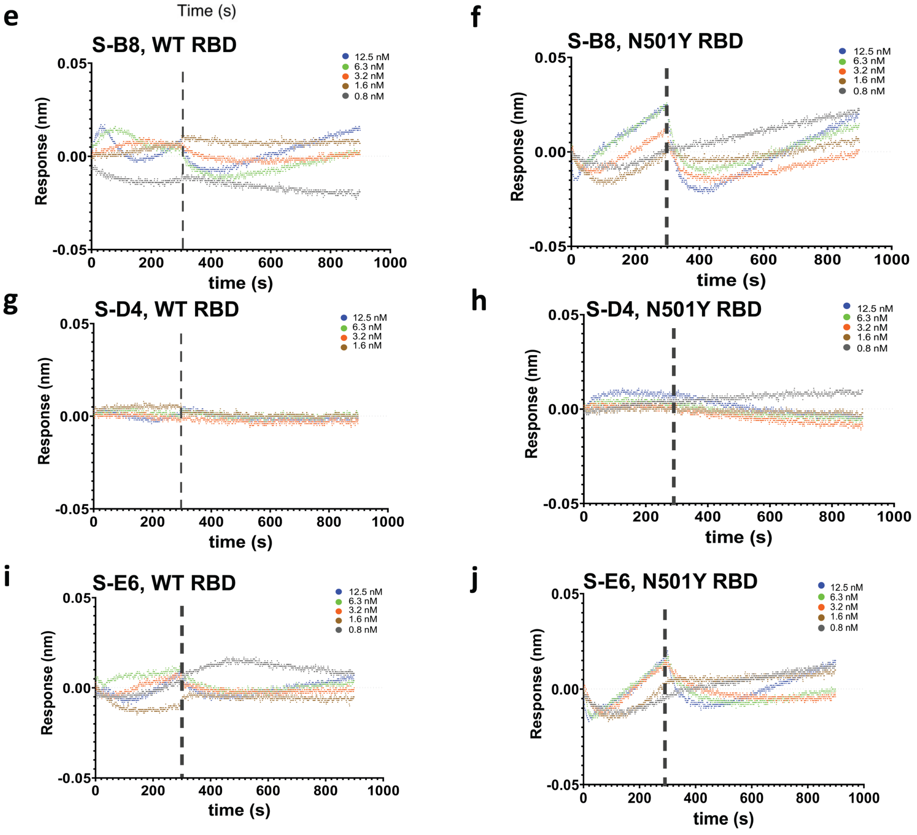

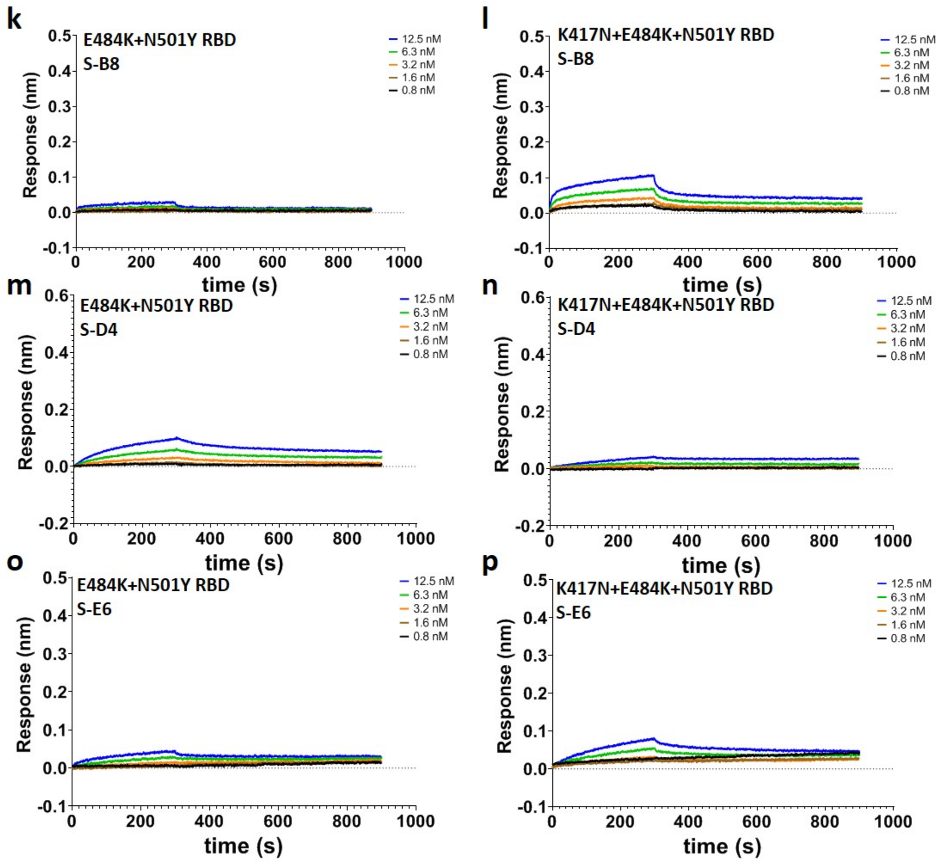

Figure S4. Binding kinetics of antibodies to SARS-CoV-2 RBD and mutants. a, b, c) Kinetics characterization of antibody Fab binding to wild-type RBD was measured by biolayer interferometry (BLI). Real time association and disassociation binding signals (solid lines) and model fitting (dashed lines) for S-B8 Fab (a), S-D4 Fab (b) or S-E6 (c) were plotted. Gray vertical dashed lines indicate the separation between association and disassociation steps. Antibody Fabs were immobilized on the Fab2G biosensor and titrated with serially diluted RBD in solution with the concentrations indicated by colored lines. A 1:1 binding model was used for the Fab data fitting. d) The association-rate (k_{on}), dissociation-rate (k_{off}), dissociation constant (K_D), and R^2 value for the fitting are shown. e-j) Residual plot of model fitting. Fitting residuals between fitting model and real-time association and disassociation of S-B8 (e), S-D4 (g) or S-E6 (i) to wild-type SARS-CoV-2 RBD (Figure 3b-d) are shown. Residuals between binding curves (Figure 3e-g) and fitting curves of S-B8 (f), S-D4 (h) or S-E6 (j) to N501Y S-RBD are also plotted. Gray vertical dashed lines indicate the separation between association and disassociation steps. k, m, o) Binding kinetics of IgG antibodies with E484K+N501Y S-RBD were measured by biolayer interferometry (BLI).

Biotinylated S-RBD mutant was loaded onto the SA biosensor for detection of binding kinetics with S-B8 (k) and S-E6 (o), while S-RBD amine coupled to AR2G sensor was utilized for S-D4 (m), with detection on the Octet. l, n, p) Binding kinetics of antibodies with K417N+E484K+N501Y S-RBD were also measured by biolayer interferometry (BLI). The binding curves of S-B8 (l), S-D4 (n) and S-E6 (p) are shown.

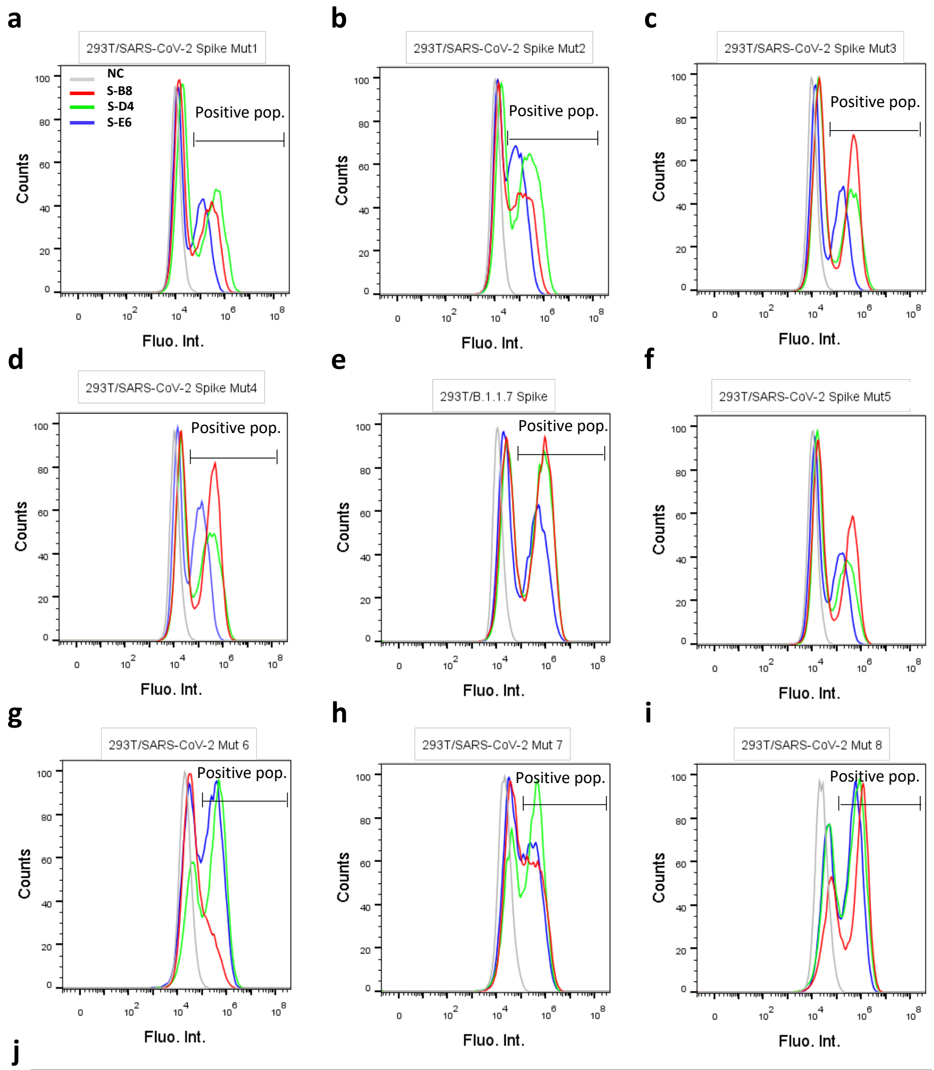

j

Ab.	Mut1 D215H	Mut2 S247R	Mut3 D614G	Mut4 N439K+ D614G	B.1.1.7	Mut5 N501Y+ D614G	Mut6 E484K+ N501Y+ D614G	Mut7 K417N+ E484K+N501Y +D614G	Mut8 K417N+ N501Y+ D614G
NC	40769	40137	37819	52471	40177	37107	66323	62810	107064
S-B8	268724	194415	392330	363935	724625	356658	70733	58004	335733
S-D4	410952	334880	414362	360133	714938	281372	137717	147482	154863
S-E6	135268	109148	156969	133012	418167	153050	138815	140947	311657

Figure S5. FACS analysis of antibody binding to cell surface-expressed mutated SARS-CoV-2 spike protein. HEK293T cells transfected with expression plasmid encoding the mutated full-length spike of SARS-CoV-2 were incubated with the three *h*ACE2 competitive IgG4 antibodies. The cells were then stained with FITC labeled anti-human IgG Fc secondary antibody and analyzed by FACS. Cells stained with only secondary antibody were set as negative control (NC). Positive binding cells populations were labeled as positive pop. a) Mut1: D215H, b) Mut2: S247R,

c) Mut3: D614G, d) Mut4: N439K+D614G, e) Alpha variant (B.1.1.7): spike, f) Mut5: N501Y+D614G, g) Mut 6: N501Y+E484K+D614G, h) Mut 7: K417N+E484K+N501Y+D614G, i) Mut 8: K417N+N501Y+D614G. Grey line: NC, Red line: S-B8, Green line: S-D4, Blue line: S-E6. j) Mean Fluorescent Intensity of Abs to each mutant is listed.

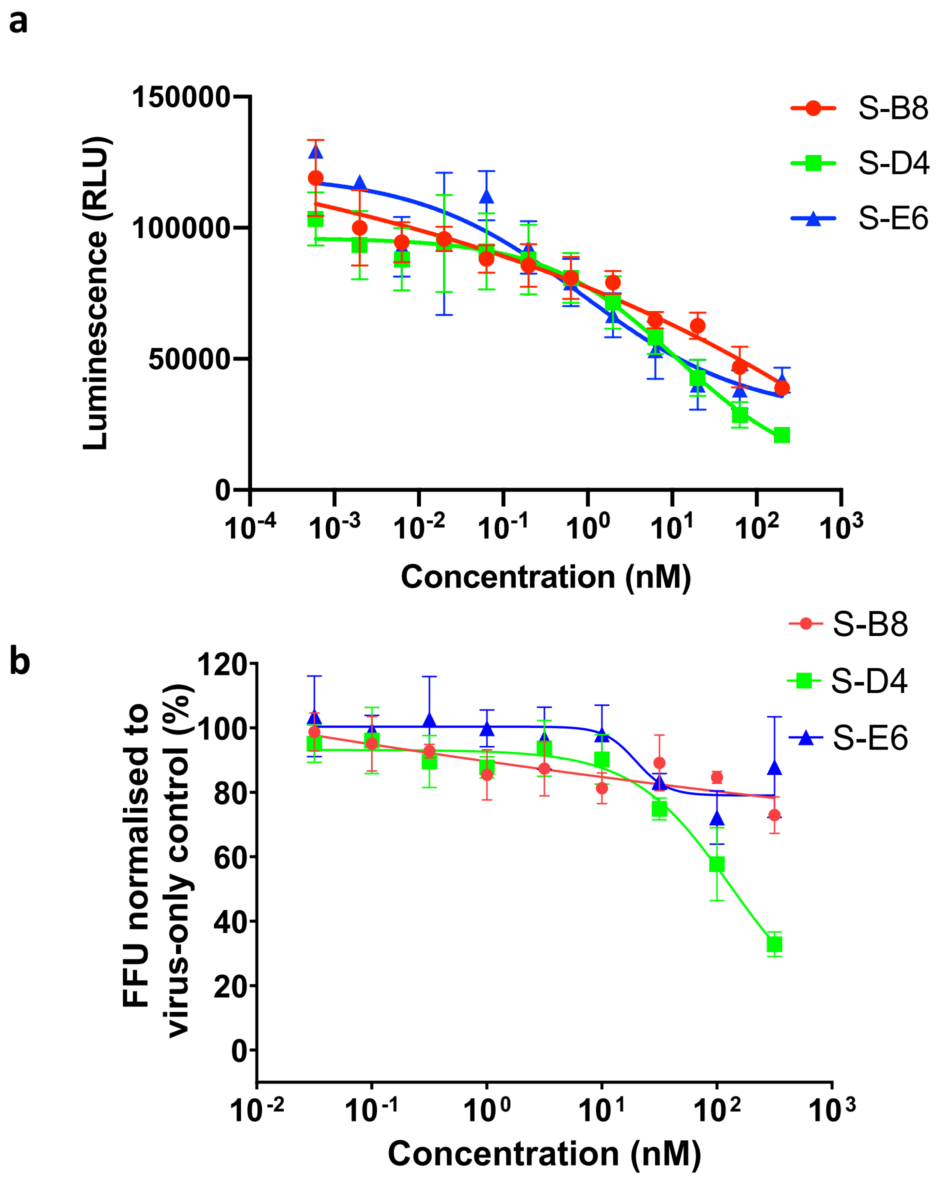

Figure S6. Neutralization of antibodies on K417N+E484K mutant and beta variant (B.1.351). a) Neutralization ability of the three *h*ACE2 competitive antibodies to K417N+E484K+N501Y SARS-CoV-2 pseudovirus was tested. b) A microneutralization assay was adopted for testing of the three antibodies on the authentic virus of beta variant.

```
            1       10        20        30        40        50        60        70        80        90
IGHV3-53*01 EVQLVESGGGLIQPGGSLRLSCAASGFTVSSNYMSWVRQAPGKGLEWVSVIYSGGSTYYADSVKGRFTISRDNSKNTLYLQMNSLRAEDTAVYYCAR
IGHV3-53*02 EVQLVETGGGLIQPGGSLRLSCAASGFTVSSNYMSWVRQAPGKGLEWVSVIYSGGSTYYADSVKGRFTISRDNSKNTLYLQMNSLRAEDTAVYYCAR
IGHV3-53*03 EVQLVESGGGLIQPGGSLRLSCAASGFTVSSNYMSWVRQPPGKGLEWVSVIYSGGSTYYADSVKGRFTISRDNSKNTLYLQMNSLRAEDTAVYYCAR
IGHV3-53*04 EVQLVESGGGLVQPGGSLRLSCAASGFTVSSNYMSWVRQAPGKGLEWVSVIYSGGSTYYADSVKGRFTISRHNSKNTLYLQMNSLRAEDTAVYYCAR
IGHV3-53*05 EVQLVETGGGLIQPGGSLRLSCAASGFTVSSNYMSWVRQAPGKGLEWVSVIYSGGSTYYADSVKGRFTISRDNSKNTLYLQMNSLRAEDTAVYYCAR
IGHV3-66*01 EVQLVESGGGLVQPGGSLRLSCAASGFTVSSNYMSWVRQAPGKGLEWVSVIYSGGSTYYADSVKGRFTISRDNSKNTLYLQMNSLRAEDTAVYYCAR
IGHV3-66*02 EVQLVESGGGLVQPGGSLRLSCAASGFTVSSNYMSWVRQAPGKGLEWVSVIYSGGSTYYADSVKGRFTISRDNSKNTLYLQMNSLRAEDTAVYYCAR
IGHV3-66*03 EVQLVESGGGLIQPGGSLRLSCAASGFTVSSNYMSWVRQAPGKGLEWVSVIYSCGSTYYADSVKGRFTISRDNSKNTLYLQMNSLRAEDTAVYYCAR
IGHV3-66*04 EVQLVESGGGLVQPGGSLRLSCAASGFTVSSNYMSWVRQAPGKGLEWVSVIYSGGSTYYADSVKGRFTISRDNSKNTLYLQMNSLRAEDTAVYYCAR
```

Figure S7. Sequence alignment of IGHV3-53 and IGHV3-66. Germline sequences from all known alleles are analyzed. Positions with any amino-acid variation are shown in red.

```
              V Q L V Q S G G G L I Q P G G S L R L S C A A S G F T V S
S-B8 HC       AGGTTCAGCTGGTACAGTCTGGAGGAGGCTTGATCCAGCCGGGGGGTCCCTGAGACTCTCCTGTGCAGCCTCTGGGTTCACCGTCAGTC
IGHV3-66*03   ....G........GG.........................T...............................................A
              V Q L V E S G G G L I Q P G G S L R L S C A A S G F T V S

              L S H M N W V R Q A P G K G L E W V S I T Y G D G N S D Y A
S-B8 HC       TCTCCCACATGAACTGGGTCCGCCAGGCTCCAGGGAAGGGACTGGAGTGGGTCTCAATTACTTATGGCGATGGTAACTCAGACTATGCAG
IGHV3-66*03   G.AA.T......G...........................G...............G...T....A..TG.....G.A..T....C....
              S N Y M S W V R Q A P G K G L E W V S V I Y S C G S T Y Y A
              CDR1                                  CDR2

              D S V K G R F T I S R D N S K N T L Y L Q M N S L R A E D T
S-B8 HC       ACTCCGTGAAGGGCCGATTCACCATCTCCAGAGACAATTCCAAGAACACGCTGTATCTGCAAATGAACAGCCTGAGAGCTGAGGACACGG
IGHV3-66*03   ..........................................................T...............................
              D S V K G R F T I S R D N S K N T L Y L Q M N S L R A E D T
              CDR2

              A V Y Y C A R E Y Y Y G M D V W G Q G T T V T V S S
S-B8 HC       CTGTGTATTACTGTGCGAGAGAATACTACTACGGTATGGACGTCTGGGGCCAAGGGACCACGGTCACCGTCTCCTCAG
IGHV3-66*03   .....................---------------------------------------------------------
              A V Y Y C A R
                           CDR3

              D I V M T Q S P D S L A V S L G E R A T I N C K S S Q S V L
S-B8 KC       GACATCGTGATGACCCAGTCTCCAGACTCCCTGGCTGTGTCTCTGGGCGAGAGGGCCACCATCAACTGCAAGTCCAGCCAGAGTGTTTTA
IGKV4-1*01    ..........................................................................................
              D I V M T Q S P D S L A V S L G E R A T I N C K S S Q S V L
                                                             CDR1

              Y S S N N K N Y L A W Y Q Q K P G Q P P K L L I Y W A S T R
S-B8 KC       TACAGCTCCAACAATAAGAACTACTTAGCTTGGTACCAGCAGAAACCAGGACAGCCTCCTAAGCTGCTCATTTACTGGGCATCTACCCGG
IGKV4-1*01    ..........................................................................................
              Y S S N N K N Y L A W Y Q Q K P G Q P P K L L I Y W A S T R
              CDR1                                              CDR2

              E S G V P D R F S G S G S G T D F T L T I S S L Q A E D V A
S-B8 KC       GAATCCGGGGTCCCTGACCGATTCAGTGGCAGCGGGTCTGGGACAGATTTCACTCTCACCATCAGCAGCCTGCAGGCTGAAGATGTGGCA
IGKV4-1*01    ..........................................................................................
              E S G V P D R F S G S G S G T D F T L T I S S L Q A E D V A
              CDR2

              V Y Y C Q Q Y Y S L P L T F G G G T K L E I K
S-B8 KC       GTTTATTACTGTCAACAATATTATAGTCTTCCTCTCACTTTCGGCGGAGGGACCAAGCTGGAGATCAAA
IGKV4-1*01    ..............G............AC.....-----------------------------------
              V Y Y C Q Q Y Y S T P
                      CDR3

              E S G P G L V K P S E T L S L T C A V S G G S L S S V N Y Y
S-E6 HC       GGAGTCTGGTCCAGGACTGGTGAAGCCTTCGGAGACCCTGTCCCTCACCTGCGCTGTCTCTGGTGGCTCTCTCAGCAGTGTTAATTACTA
IGHV4-31*11   ......G..C....................AC.....................................CA.........G.GG......
              E S G P G L V K P S Q T L S L T C A V S G G S I S S G G Y Y
                                                              CDR1

              W S W I R Q H P G K G L E W I G Y I Y Y S G S T N Y N P S L
S-E6 HC       CTGGAGCTGGATCCGCCAGCACCCAGGGAAGGGCCTGGAGTGGATTGGGTACATCTATTACAGTGGGAGTACCAACTACAACCCGTCCCT
IGHV4-31*11   .....................................................................C...T................
              W S W I R Q H P G K G L E W I G Y I Y Y S G S T Y Y N P S L
              CDR1                                  CDR2

              K S R V T M S L D T S K N Q F S L K L S S V T A A D T A V Y
S-E6 HC       CAAGAGTCGAGTCACCATGTCACTGGACACGTCCAAGAACCAGTTCTCCCTGAAACTGAGCTCTGTGACTGCCGCGGACACGGCCGTCTA
IGHV4-31*11   ............T.....A...G.A........T....................G................................G..
              K S R V T I S V D T S K N Q F S L K L S S V T A A D T A V Y
              CDR2

              Y C A T P G A I M G A L H I W G Q G T L V T V S S
S-E6 HC       TTACTGTGCGACCCCCGGAGCTATTATGGGTGCTCTTCATATCTGGGGCCAAGGCACCCTGGTCACCGTCTCCTCAG
IGHV4-31*11   ...........------------------------------------------------------------------
              Y C A
                        CDR3

              Q A V L T Q P S S A S S T P G Q R V I I S C S G S S S N I G
S-E6 LC       CAGGCTGTGCTCACTCAGCCGTCCTCGGCGTCCTCGACCCCCGGGCAGAGGGTCATCATCTCTTGTTCTGGGAGCAGCTCCAATATCGGG
IGLV1-44*01   ...T.......G........AC....A.....TGG....................C...............A...........C.....A
              Q S V L T Q P P S A S G T P G Q R V T I S C S G S S S N I G
                                                          CDR1

              S N T V S W Y Q Q V P G A A P K L L I Y F D Y R R P S G V P
S-E6 LC       AGTAACACTGTCAGCTGGTACCAGCAGGTCCCAGGAGCGGCCCCCAAACTCCTCATCTACTTTGATTATCGACGTCCCTCAGGGGTCCCT
IGLV1-44*01   .....T.....A.A.............C........A......................TAG.A..A...AG..G...............
              S N T V N W Y Q Q L P G T A P K L L I Y S N N Q R P S G V P
              CDR1                                     CDR2

              D R F S G T R S G T S A S L G I S G L Q S E D E A D Y Y C A
S-E6 LC       GACCGCTTCTCTGGCACCAGGTCTGGCACCTCTGCCTCCCTGGGCATCAGTGGGCTCCAGTCTGAGGATGAGGCTGATTATTACTGTGCC
IGLV1-44*01   .....A.........T...A............A..........C.............................................A
              D R F S G S K S G T S A S L A I S G L Q S E D E A D Y Y C A
                                                                       CDR3

              A W D D S L S A W V F G R G T K L T V L
S-E6 LC       GCATGGGATGACAGCCTGAGTGCTTGGGTGTTCGGCAGAGGGACCAAGCTGACCGTCCTAG
IGLV1-44*01   ...................A..---------------------------------------
              A W D D S L N
              CDR3
```

Figure S8. Germline sequences and somatic hypermutation (SHM) of S-B8 and S-E6. Germline sequences are aligned to heavy and light chain sequences for each antibody. Residues from SHM are colored in magenta and antibody residues interacting with SARS-CoV-2 S-RBD are boxed.

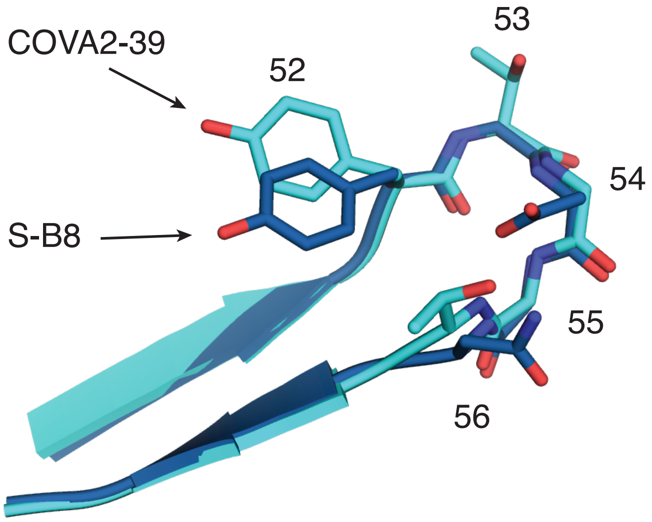


```
              52  56
COVA2-39      YTGGT
S-B8          YGDGN
```

Figure S9. Structural comparison of CDRH2 in S-B8 and COVA2-39 (PDB 7JMP). Residues in the β-turn region at the apex of CDRH2 are shown as sticks with corresponding sequences shown on the right and sequence numbers labeled.

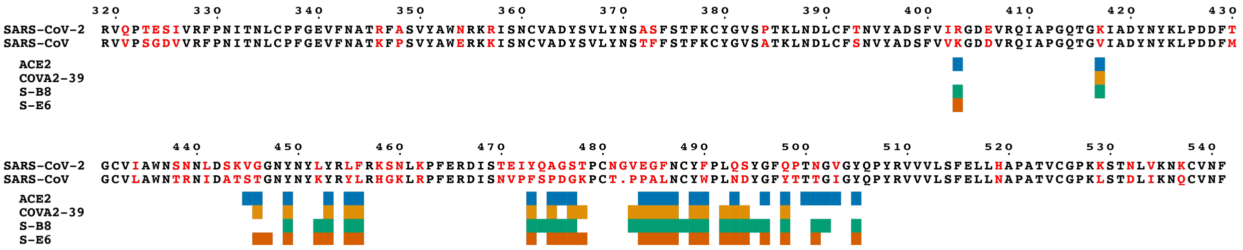

Figure S10. Fab epitopes and *h*ACE2 binding site. SARS-CoV-2 S-RBD epitopes of S-B8, S-E6 and COVA2-39 (PDB 7JMP) as well as *h*ACE2 binding site on the S-RBD were analyzed with PISA program using buried surface area (BSA)>0 Å^2 as the criterion. Each antibody epitope or *h*ACE2 binding site is shown as blocks below the S-RBD sequence under the corresponding residue position. The sequences of SARS-CoV-2 and SARS-CoV S-RBD are aligned and shown on top of the epitope (colored blocks) for comparison of sequence variation at the epitope sites. Numbers on top of the sequence indicate the amino-acid positions on the SARS-CoV-2 S-RBD.

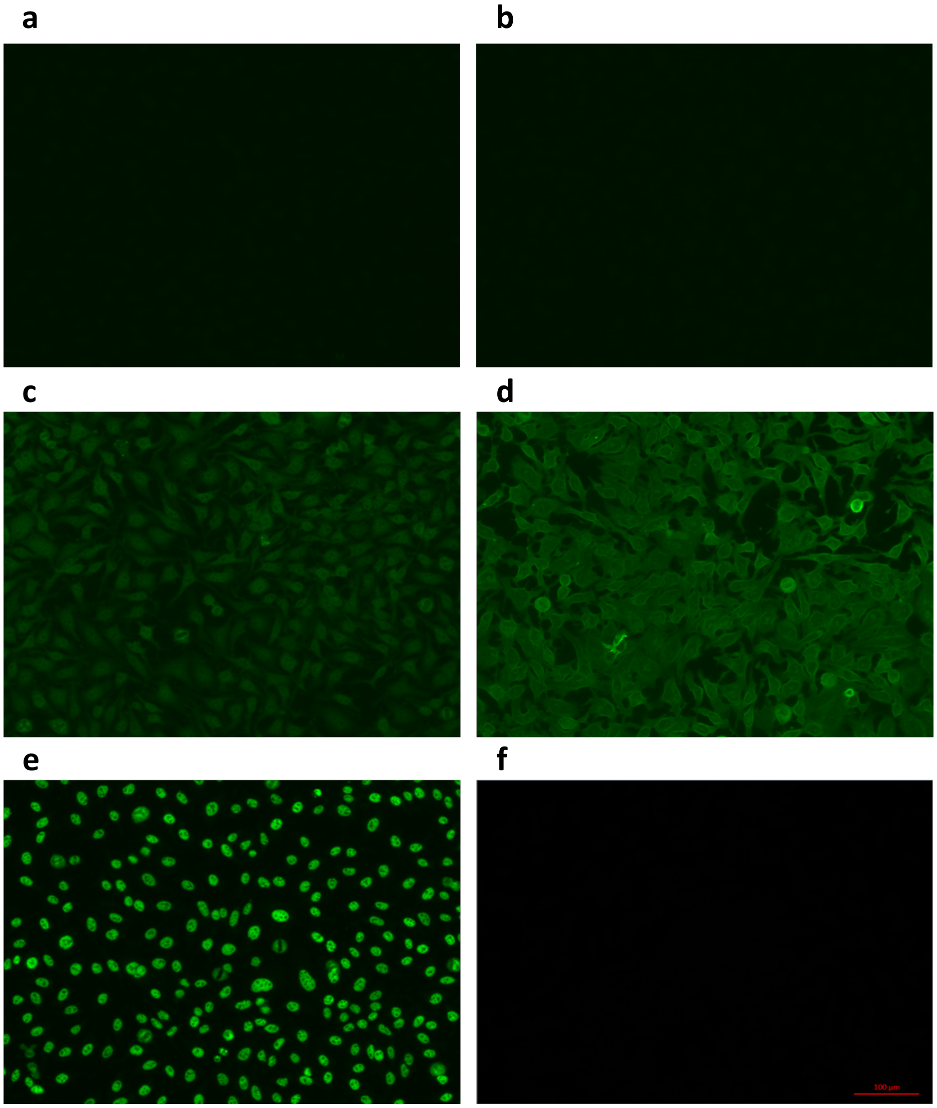

Figure S11. Autoreactivity detection of selected antibodies. Autoreactivity of S-D4 (a), S-E6 (b), S-B8 (c) and the S-B8 putative germline antibody (d) were tested by using an HEp-2 cell based antinuclear-antibody kit. The S-B8 putative germline antibody was generated by mutating the SHMs of S-B8 back to the germline sequence of IGHV3-66 as shown in Figure S8. Positive control (PC) (e) and negative control (NC) (f) are from serum of patients with or without autoimmune disease, which is included in the kit. The green color indicates positive binding of the tested antibodies to HEp-2 cells. Bar=100 μm.

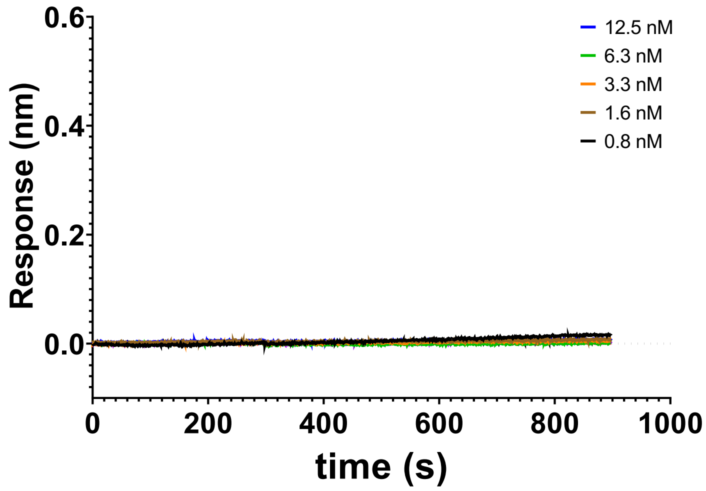

Figure S12. Binding kinetics of S-B8 putative germline antibody to the spike protein. Binding kinetics were measured by biolayer interferometry (BLI). Biotinylated S-RBD was loaded onto the SA biosensor for detection of binding kinetics with the S-B8 putative germline antibody.

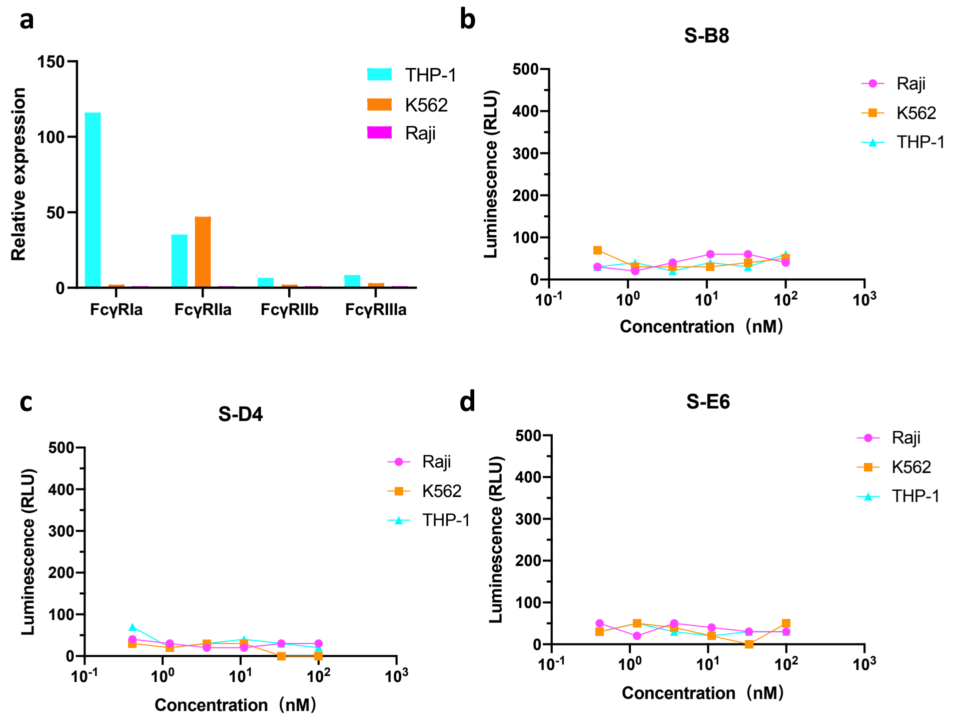

Figure S13. ADE activity assessment of the three *h*ACE2 competitive antibodies using SARS-CoV-2 pseudovirus. a) mRNA level of the Fcγ receptors expression in THP-1, K562 and Raji cells. b-d) ADE activity of S-B8, S-D4 and S-E6 on the three cell lines. The pseudovirus infection assay was performed as described in methods section, except that the *h*ACE2 overexpressed cells were replaced by Raji, K562 and THP-1 cells. Pseudoviruses pre-incubated with serially diluted antibody mixtures were added to Raji, K562, and THP-1 cells to evaluate their ability to enhance infection.

a

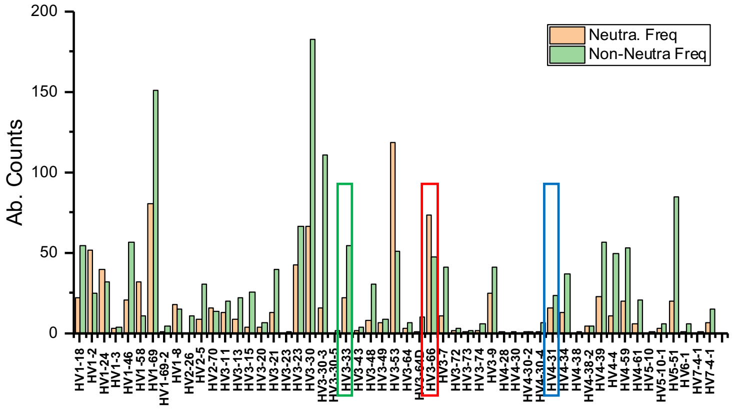

b

	VH germline
S-B8	IGHV3-66*03
S-D4	IGHV3-33*01
S-E6	IGHV4-31*11

Figure S14. IGHV germline distribution of SARS-CoV-2 spike-targeting antibodies. a) IGHV distribution of 2,438 SARS-CoV-2 binding antibodies reported in the public database (http://opig.stats.ox.ac.uk/webapps/covabdab/) were analyzed as of July 24, 2021. The numbers of neutralizing and non-neutralizing antibodies of certain germlines were also calculated and shown. b) The IGHV of three competitive antibodies are indicated and shown in different colors in the Table and in **a**.

Table S1. Crystallographic data collection and refinement statistics

	S-B8 + RBD	S-E6 + RBD
**Data collection**		
Beamline	APS 23ID-B	APS 23ID-B
Wavelength (Å)	1.03317	1.03373
Space group	*R* 1 3 1	*C* 1 2 1
Unit cell parameters		
a, b, c (Å)	191.6, 191.6, 117.4	242.1, 70.2, 91.9
α, β, γ (°)	90, 90, 120	90, 108.5, 90
Resolution (Å) [a]	50.0-2.25 (2.30-2.25)	50.0-2.70 (2.75-2.70)
Unique reflections [a]	74,079 (3,622)	37,826 (1,647)
Redundancy [a]	6.4 (2.6)	3.5 (2.6)
Completeness (%) [a]	97.4 (71.4)	93.3 (82.8)
$\langle I/\sigma_I \rangle$ [a]	17.3 (1.4)	12.5 (1.7)
R_{sym}[b] (%) [a]	9.8 (44.6)	9.2 (56.0)
R_{pim}[b] (%) [a]	4.0 (28.5)	5.5 (38.0)
$CC_{1/2}$[c] (%) [a]	99.4 (77.5)	98.4 (72.0)
**Refinement statistics**		
Resolution (Å)	47.9-2.25	49.3-2.70
Reflections (work)	70,217	33,627
Reflections (test)	3,821	1,791
R_{cryst}[d] / R_{free}[e] (%)	17.9/22.1	23.6/28.3
No. of atoms	10,193	8,955
Macromolecules	9,665	8,927
Glycans	28	28
Solvent	500	-
Average *B*-value (Å^2)	42	47
Macromolecules	42	47
Fab	40	43
RBD	46	57
Glycans	65	113
Solvent	45	-
Wilson *B*-value (Å^2)	37	44
**RMSD from ideal geometry**		
Bond length (Å)	0.005	0.004
Bond angle (°)	0.78	0.67
**Ramachandran statistics (%)**		
Favored	97.8	95.5
Outliers	0.0	0.2
**PDB code**	7KN3	7KN4

[a] Numbers in parentheses refer to the highest resolution shell.
[b] $R_{sym} = \Sigma_{hkl} \Sigma_i | I_{hkl,i} - \langle I_{hkl} \rangle | / \Sigma_{hkl} \Sigma_i I_{hkl,i}$ and $R_{pim} = \Sigma_{hkl} (1/(n-1))^{1/2} \Sigma_i | I_{hkl,i} - \langle I_{hkl} \rangle | / \Sigma_{hkl} \Sigma_i I_{hkl,i}$, where $I_{hkl,i}$ is the scaled intensity of the ith measurement of reflection h, k, l, $\langle I_{hkl} \rangle$ is the average intensity for that reflection, and *n* is the redundancy.
[c] $CC_{1/2}$ = Pearson correlation coefficient between two random half datasets.
[d] $R_{cryst} = \Sigma_{hkl} | F_o - F_c | / \Sigma_{hkl} | F_o |$ x 100, where F_o and F_c are the observed and calculated structure factors, respectively.
[e] R_{free} was calculated as for R_{cryst}, but on a test set comprising 5% of the data excluded from refinement.

Table S2. Hydrogen bonds and salt bridges identified at the antibody-RBD interface using the PISA program*

Chain	Residue	Atom	Distance (Å)	Chain	Residue	Atom
S-B8				SARS-CoV-2 RBD		
Hydrogen bonds						
H	GLY 26	N	3.8	A	TYR 449	OH
H	THR 28	N	2.9	A	GLN 493	OE1
H	THR 28	N	3.4	A	SER 494	OG
H	THR 28	OG1	2.5	A	SER 494	OG
H	GLY 53	N	2.9	A	GLU 484	OE1
H	ASP 54	N	3.7	A	GLU 484	OE2
H	ASN 76	ND2	3.3	A	TYR 449	OH
H	ALA 24	O	3.9	A	TYR 449	OH
H	LEU 31	O	3.7	A	GLN 493	NE2
H	SER 32	OG	3.0	A	GLN 493	NE2
H	GLU 95	O	2.8	A	TYR 489	OH
L	TYR 32	OH	2.7	A	ALA 475	O
L	TYR 27d	OH	3.7	A	SER 477	O
L	TYR 27d	OH	3.7	A	GLN 474	NE2
L	TYR 91	O	3.2	A	ASN 487	ND2
Salt bridges						
H	HIS 33	NE2	3.1	A	GLU 484	OE1
S-E6				SARS-CoV-2 RBD		
Hydrogen bonds						
H	ASN 33	ND2	2.9	A	ALA 475	O
H	ALA 97	N	2.8	A	ASN 487	OD1
H	TYR 34	OH	2.9	A	ASN 487	ND2
L	ARG 53	NH2	2.7	A	PHE 490	O
L	ARG 53	NH2	3.0	A	LEU 492	O
L	ARG 53	NE	3.7	A	GLN 493	OE1
L	TYR 52	OH	3.0	A	GLN 493	OE1
L	ARG 53	NH2	3.4	A	GLN 493	OE1
L	GLY 68	N	3.3	A	ASN 501	OD1
L	SER 27	OG	2.2	A	TYR 505	OH
L	GLY 29	O	3.1	A	ARG 403	NH1
L	GLY 29	O	3.2	A	ARG 403	NH2
L	PHE 50	O	3.2	A	GLN 493	NE2
L	ASP 51	OD2	3.0	A	GLN 493	NE2
L	TYR 52	OH	2.7	A	SER 494	N
L	SER 67	O	3.6	A	ASN 501	ND2
Salt bridges						
L	ARG 53	NH1	3.6	A	GLU 484	OE1
L	ARG 53	NE	4.0	A	GLU 484	OE2
L	ARG 53	NH1	2.5	A	GLU 484	OE2

* Somatically hypermutated residues are highlighted in yellow.
